# Co-occurrence patterns and related risk factors of ischemic heart disease and type 2 diabetes in burden of disability-adjusted life years among people aged 55 years and older across 203 countries and territories

**DOI:** 10.3389/fendo.2025.1693166

**Published:** 2025-11-27

**Authors:** Yuting Luo, Xiaohan Qiu, Ben Hu, Haozhong Sun, Yuwei Wang, Ke Meng, Jun Gu, Likun Ma, Jing Zhang

**Affiliations:** 1 Department of Cardiology, The Second People’s Hospital of Hefei, The Affiliated Hefei Hospital of Anhui Medical University, Hefei, China; 2 Department of Cardiology, Shanghai Ninth People’s Hospital, Shanghai Jiaotong University School of Medicine, Shanghai, China; 3 Department of Cardiology, The First Affiliated Hospital of University of Science and Technology of China, Division of Life Sciences and Medicine, University of Science and Technology of China, Hefei, China

**Keywords:** ischemic heart disease, type 2 diabetes mellitus, co-occurrence patterns, risk factors, people aged 55 years and older, global

## Abstract

**Background:**

Ischemic heart disease (IHD) and type 2 diabetes mellitus (T2DM) are leading causes of disability-adjusted life years globally among adults aged 55 years and older. Although both diseases share common risk factors and pathophysiological pathways, previous research has predominantly addressed these conditions in isolation. The co-occurrence patterns and regional variations of IHD and T2DM burden remain poorly understood. We aimed to characterize the global co-occurrence patterns of IHD and T2DM from a spatial perspective and to identify the corresponding risk factors distinguishing different burden regions.

**Methods:**

Using data from the Global Burden of Diseases, Injuries, and Risk Factors Study (GBD) 2021 database, we extracted age-standardized disability-adjusted life year (DALY) rates for IHD and T2DM among individuals aged 55 years and older from 204 countries and territories. Based on quartile distributions of global DALY rates for both diseases, we classified countries into four distinct burden regions: Low-Burden Regions (56 countries), T2DM-Dominant Regions (46 countries), IHD-Dominant Regions (46 countries), and Dual-Burden Regions (56 countries). We examined temporal trends from 1990-2021, computed population attributable fractions for major risk factors, and used machine learning-based SHAP (Shapley Additive Explanations) analysis to screen and quantify the effects of corresponding risk factors distinguishing regional classifications.

**Results:**

Dual-Burden Regions were distributed across multiple geographic areas including the Caribbean and Central America, Persian Gulf states, Balkan Peninsula, Southeast Asia, West Africa, Eastern Mediterranean, and Northern Europe. The spatial distribution revealed distinct geographic clustering, with higher IHD rates in Eastern Europe and Central Asia, and elevated T2DM rates in Pacific Island nations and parts of the Middle East. Countries and territories with the highest burden for both diseases included North African countries (eg, Morocco: IHD 25,193.1/100,000 and T2DM 32,197.24/100,000) and Pacific Island nations such as Fiji exhibiting IHD burden of 24,758.17 per 100,000 and T2DM burden of 32,197.24 per 100,000. Marshall Islands showed IHD burden of 25,107.72/100,000 and T2DM burden of 22,122.46/100,000, while Nauru demonstrated the highest IHD burden (39,483.92/100,000). High systolic blood pressure contributed most to IHD burden globally (49.79%), while high body-mass index dominated T2DM burden (51.89%). Environmental factors demonstrated clear regional gradients, with household air pollution ranging from 4·58% in Low-Burden to 14.43% in Dual-Burden Regions for IHD. High body-mass index contributed 51.89% to T2DM burden globally, with regional variation from 40.61% in IHD-Dominant to 51.36% in Low-Burden Regions. SHAP analysis identified sociodemographic index (SDI2021) as the primary factor distinguishing Low-Burden from Dual-Burden Regions for both IHD (mean |SHAP| = 1.245) and T2DM (mean |SHAP| = 1.317). Diet high in processed meat consistently showed strong discriminatory power across multiple regional comparisons for T2DM (SHAP values 0.923-1.721), while secondhand smoke emerged as a critical differentiator with SHAP values exceeding 1.0 across various regional distinctions. Diet low in vegetables served as a primary differentiator between Low-Burden and T2DM-Dominant Regions (mean |SHAP| = 1.188).

**Conclusion:**

The co-occurrence of IHD and T2DM exhibits pronounced global heterogeneity, with Pacific Island nations and multiple geographic regions including Gulf states, North Africa, and other areas bearing disproportionate dual-burden. Socioeconomic development level fundamentally characterizes dual-burden status, while dietary and environmental factors serve as key regional differentiators. Intervening in modifiable risk factors, particularly processed meat consumption, vegetable intake, and environmental exposures, can fundamentally reduce the global burden of these co-occurring diseases.

## Introduction

Ischemic heart disease (IHD) is the leading cause of mortality and disability globally, while type 2 diabetes mellitus (T2DM) represents one of the fastest-growing health challenges worldwide (1, 2). These conditions present serious health hazards and impose substantial healthcare burdens, particularly in low- and middle-income countries where healthcare infrastructure struggles to manage the dual burden (1, 3, 4). Without effective interventions, the worldwide burden owing to these two conditions is projected to increase substantially by 2050, threatening global health security and economic stability (1, 5–8). The COVID-19 pandemic has further highlighted the vulnerability of individuals with these conditions, as patients with IHD and T2DM demonstrated significantly higher mortality risks (9–11). Considering that most of the disease burden stems from modifiable risk factors, research on these factors is of paramount importance for preventing both diseases and achieving Sustainable Development Goal targets.

Substantial evidence suggests that IHD and T2DM share common risk factors and pathophysiological mechanisms, including endothelial dysfunction, chronic inflammation, insulin resistance, and accelerated atherosclerosis (12, 13). Clinically, risk factors such as hypertension, dyslipidemia, and obesity influence both conditions significantly. Environmental factors including air pollution, dietary patterns influenced by globalization, and behavioral factors also contribute substantially to disease development (13–15). T2DM patients demonstrate a 2-4-fold increased risk of developing IHD, while IHD patients frequently develop glucose intolerance (16, 17). However, most studies examine IHD and T2DM separately or focus on limited geographic regions, failing to capture the global complexity of disease co-occurrence patterns (1, 16). Climate change, urbanization, and recent geopolitical tensions have introduced additional complexity, as these factors disproportionately affect populations already vulnerable to these conditions. Global-scale, high-quality research on the co-occurrence of IHD and T2DM remains limited due to substantial challenges in data accessibility and analytical methods, despite the urgent need for evidence-based strategies to achieve the World Health Assembly’s commitment to reducing premature mortality from non-communicable diseases by one-third by 2030 (18).

The objective of this study was to investigate the co-occurrence of IHD and T2DM from a spatial perspective, using the latest data available from the Global Burden of Disease, Injuries, and Risk Factors Study (GBD) 2021 database. Through systematic exploration across 204 countries and territories, we aimed to uncover global co-occurrence patterns, classify regions based on their disease burden profiles, examine temporal trends from 1990 to 2021, and identify corresponding key risk factors using advanced machine learning techniques including Shapley Additive exPlanations (SHAP) analysis. Precisely pinpointing these risk factors and co-occurrence patterns at the country level can offer guidance and evidence-based support for tailored prevention and control strategies. This study aims to inform the development of tailored prevention and control strategies, with the goal of reducing the incidence of IHD and T2DM and alleviating the associated global disease burden.

## Methods

### Data source

The data utilized in this study were extracted from the Global Burden of Disease Study 2021 (GBD 2021) (GHDx: https://vizhub.healthdata.org/gbd-results/) (19). GBD 2021 provides a comprehensive assessment of the burden attributable to 371 diseases and 88 risk factors across global, regional, and national levels from 1990 to 2021. This investigation examines global co-occurrence patterns and spatial distributions of age-standardized disability-adjusted life year (DALY) rates for IHD and T2DM, alongside their attributable risk factor burdens. Advanced statistical modeling and machine learning techniques, including age-specific age-standardized rate calculations and SHAP analysis, were employed to identify critical risk factors and quantify their contributions to disease burden. The study adheres to the Guidelines for Accurate and Transparent Health Estimates Reporting (GATHER). Ethical approval, including a waiver of informed consent due to the use of de-identified data, was granted by the Institutional Review Board at the University of Washington (https://www.healthdata.org/research-analysis/gbd).

### Disease definitions

IHD was defined as a condition of myocardial blood supply insufficiency due to coronary artery stenosis. This encompasses both acute events, characterized by clinical criteria for myocardial necrosis or abnormal cardiac biomarkers accompanied by ischemic symptoms/electrocardiographic/imaging evidence—and chronic events, including stable angina diagnosed through physician assessment or validated questionnaires. T2DM was identified using standard diagnostic criteria (fasting plasma glucose ≥126 mg/dL or ongoing glucose-lowering treatment) (20). Its non-fatal burden was estimated indirectly by subtracting the physician-diagnosed, registry/hospital-record-confirmed burden of type 1 diabetes from the overall diabetes burden. Disease burden quantification incorporated: Years Lived with Disability (YLDs), calculated as prevalence multiplied by condition-specific disability weights (with microsimulation adjustment for comorbidity effects). Years of Life Lost (YLLs), derived from mortality counts multiplied by standard life expectancy at the age of death. DALYs were computed as the sum of YLLs and YLDs, where YLDs quantify health loss from non-fatal conditions. YLLs were calculated by multiplying estimated deaths by standard life expectancy at the age of death. YLDs were determined by multiplying prevalence by disability weights (ranging from 0 [perfect health] to 1 [equivalent to death]), which represent the magnitude of health loss associated with a given disease. The aggregate DALYs within a population measure the total disease burden experienced by that population. Detailed estimation methodologies are provided in the **Supplementary Materials**.

### Definition of comorbidity patterns and regional stratification for IHD and T2MD

To examine the global co-occurrence characteristics and spatial heterogeneity of IHD and T2DM, countries/territories were stratified into four categories based on median percentiles of age-standardized DALY rates: Low-Burden Regions, T2DM-Dominant Regions, IHD-Dominant Regions, and Dual-Burden Regions. Classification criteria were defined as follows: A country/territory was categorized as a Low-Burden Region if both its IHD and T2DM age-standardized DALY rates fell below the 50th percentile globally. T2DM-Dominant Regions comprised units where T2DM DALY rates exceeded the 50th percentile, whereas IHD rates were below this threshold. IHD-Dominant Regions included units with IHD DALY rates above the 50th percentile but T2DM rates below it. Units exhibiting both IHD and T2DM DALY rates above the 50th percentile were designated Dual-Burden Regions. This stratification framework delineates four distinct geographic patterns of disease predominance, enabling systematic analysis of spatial disparities in comorbid burden.

### Risk factor selection

This study extracted age-standardized Disability-Adjusted Life Year (DALY) rates for IHD and T2DM among adults aged ≥55 years across 204 countries and territories in 2021, alongside exposure metrics for all 88 most granular risk factors encompassing environmental, occupational, and behavioral domains. We identified 27 risk factor subcategories associated with IHD and 17 subcategories linked to T2DM. Methodological details for risk factor estimation are provided in the **Supplementary Materials**. To evaluate variable importance, we implemented SHAP analysis within an XGBoost framework—a gradient-boosted tree-based ensemble machine learning algorithm. This approach quantified the differential contributions of individual risk factors to predictive outcomes, establishing a rigorous variable selection framework (21).

### Relative risk estimation

GBD 2021 generated spatiotemporally comparable RR estimates by systematically integrating RR data from randomized controlled trials, cohort studies, and case-control studies through evidence synthesis. This methodology incorporated multi-source exposure data—including Demographic and Health Surveys, censuses, ground-based and remote sensing monitoring systems, and administrative records—to construct comprehensive risk exposure assessment models. Spatiotemporal Gaussian process regression was subsequently applied to characterize exposure level distributions across geographic units and time periods, yielding rigorously standardized RR metrics (22).

### Theoretical minimum risk exposure level

The TMREL represents the counterfactual exposure distribution at which population health risk is minimized. Detailed specifications of low-risk exposure thresholds are provided in the **Supplementary Materials**.

### Population attributable fraction

The PAF is defined as the proportion of disease burden that could be reduced if exposure to a specific risk factor were lowered to the TMREL. For each risk factor, PAF was computed using the continuous exposure formula:


$$PAF=\frac{\int_{x=l}^{m}RR(x)P(x)dx-RR(x)TRMEL}{\int_{x=l}^{m}RR(x)P(x)dx}$$

where *l* denotes the minimum exposure level, *m* represents the maximum exposure level, *RR*(*x*) indicates the relative risk at exposure level *x*, TMREL signifies the counterfactual exposure distribution, and *P*(*x*) describes the current population exposure distribution. All variables were calculated with covariates stratified by age, sex, geographical location, and year. Attributable DALYs were estimated by multiplying the total DALYs for a specific outcome by its corresponding PAF, quantifying each risk factor’s proportional contribution to disease burden (23).

### Socio-demographic index

The SDI is a composite metric of development status (integrating fertility rates, educational attainment, and income per capita) that correlates with population health outcomes. SDI values range from 0 to 1, representing a theoretical development continuum relevant to health (24), with higher values indicating higher levels of socio-economic development.

### Statistical analysis

Age-standardized rates for populations aged ≥55 years were computed using the formula:


$$\frac{\sum_{i=1}^{N}\alpha_{i}W_{i}}{\sum_{i=1}^{N}W_{i}}$$

where *α_i_* denotes the age-specific rate in the *i* age group, *W_i_* represents the corresponding age group’s proportion in the GBD 2021 standard population, and *N* is the total age groups. Rates were standardized to the GBD world standard population using the ageadjust.direct function from R’s epitools package. All GBD burden estimates report 95% uncertainty intervals (UIs) encompassing the true value with 95% probability, accounting for sampling variance, model uncertainty, and data quality limitations. Average annual percentage changes (AAPC) in disease burden were quantified through joinpoint regression analysis. Machine learning implementation utilized R’s xgboost and shapviz packages for XGBoost modeling and SHAP value computation respectively. The outcome variable (Y) for the predictive models was the categorical variable representing the four disease burden regions. To precisely delineate the risk factor profiles that distinguish each disease burden pattern, we adopted a pairwise comparison framework within the XGBoost algorithm. Instead of building a single multi-class model, we constructed a series of six binary XGBoost classifiers, each designed to discriminate between two specific regions. This approach allows for a more sensitive detection of risk factors that are uniquely important in contrasting one specific pattern against another. For each of these six binary models, the input features (X) were the age-standardized exposure levels of the risk factors relevant to the diseases being compared. We subsequently performed SHAP analysis on each of the six trained XGBoost models. For a given pairwise model, the mean absolute SHAP value for each risk factor quantifies its overall importance in distinguishing between the two regions. The direction of the impact is indicated by the sign of the SHAP value for each individual observation. This methodology allowed us to generate a ranked list of the most critical risk factors driving the differences for each specific regional contrast (more details on the XGBoost model used in this study, see **Supplementary Materials**).

## Results

This study examined age-standardized DALY rates for IHD and T2DM among adults aged ≥55 years in 204 countries and territories worldwide in 2021.

### Quadrant classification and global distribution patterns

**Figure 1:**
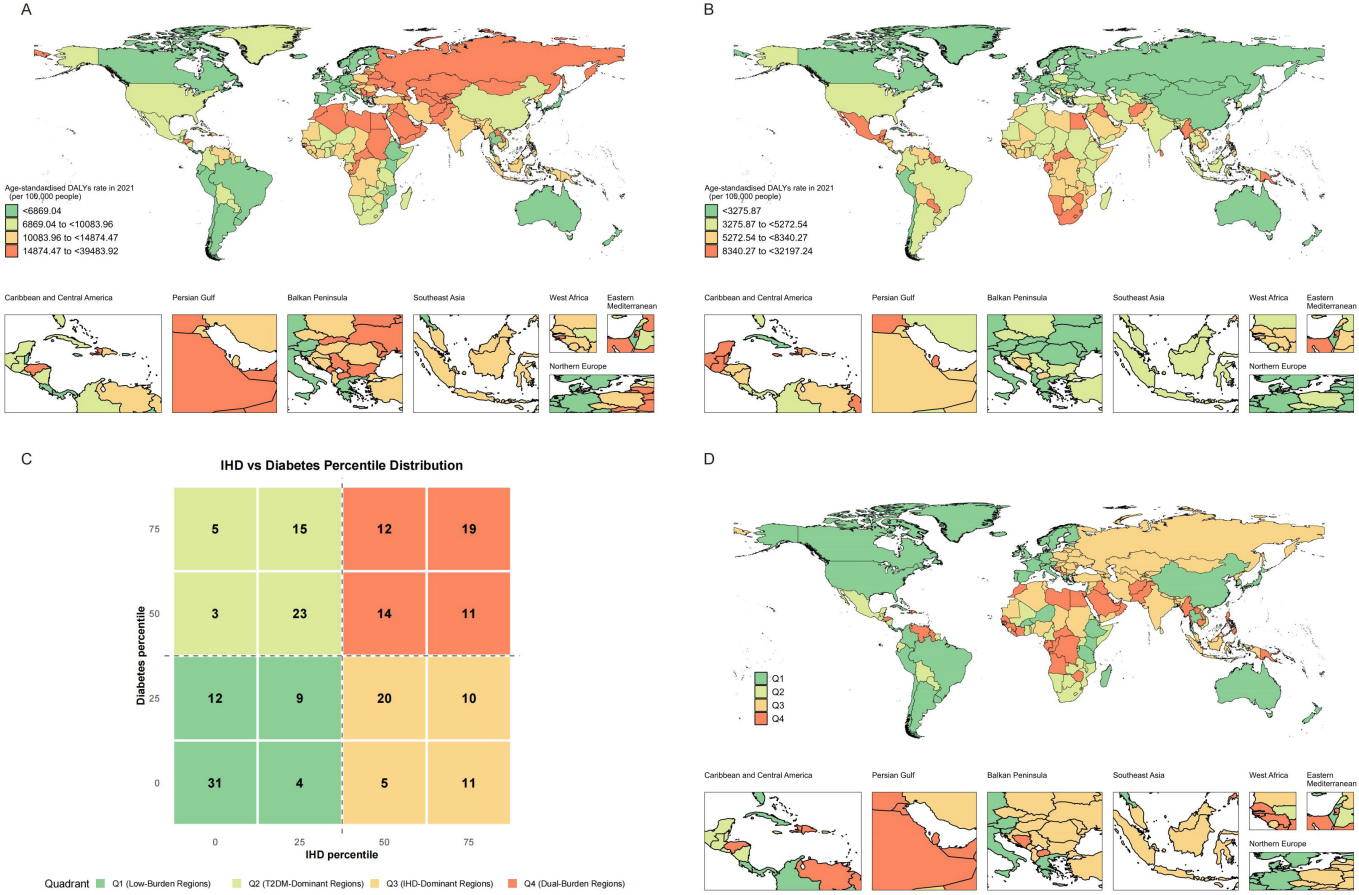
Global distribution maps showing the age-standardized disability-adjusted life years rate of IHD **(A)**, T2MD **(B)**, and characteristics **(C)** of co-occurrence in the age-standardized disability-adjusted life years rate of IHD and T2MD **(D)** among people aged 55 years and older in 2021. IHD, ischemic heart disease; T2MD, type 2 diabetes mellitus.

The overall spatial distribution of IHD and T2DM DALY rates across quartile levels exhibited distinct geographic patterns, characterized by higher IHD rates in Eastern Europe and Central Asia and elevated T2DM rates in Pacific Island nations and parts of the Middle East, with notable regional clustering of both diseases in specific geographic areas (**Figures 1A, B**). We depicted the spatial distribution of co-occurrence features of IHD and T2DM (**Figures 1C, D**). The quadrant classification based on percentile distributions revealed countries distributed across Low-Burden Regions (Q1, 56 countries [27.45%] of 204), T2DM-Dominant Regions (Q2, 46 countries [22.55%] of 204), IHD-Dominant Regions (Q3, 46 countries [22.55%] of 204), and Dual-Burden Regions (Q4, 56 countries [27.45%] of 204) (**Figure 1C**). Countries and territories with the highest burden for both diseases included North African countries (eg, Morocco: IHD 25,193.1/100,000 and T2DM 32,197.24/100,000) and Pacific Island nations (eg, Marshall Islands: IHD 25,107.72/100,000 and T2DM 22,122.46/100,000). Dual-Burden Regions were distributed across multiple geographic areas including the Caribbean and Central America, Persian Gulf states, Balkan Peninsula, Southeast Asia, West Africa, Eastern Mediterranean, and Northern Europe (**Figure 1D**).

### IHD burden patterns across four regions

Among Low-Burden Regions, substantial heterogeneity existed in IHD age-standardized DALY rates (**Supplementary Table S1**). Nordic countries demonstrated improvements across both conditions, with Denmark showing IHD decline of −5.4217% AAPC (p<0.001), Norway showing − 5.0495% AAPC (p<0.001), and Sweden showing −4.1297% AAPC (p<0.001) for IHD. High-income countries consistently demonstrated lower disease burden, with the SDI showing strong correlation with disease outcomes. T2DM-Dominant Regions demonstrated substantial heterogeneity across 46 countries and territories (**Supplementary Table S2**). Most countries in this region showed substantial IHD improvements over the study period despite their T2DM dominance, with many countries showing negative AAPC values for IHD (eg, Mauritius: IHD decline of −3.06% AAPC (p<0.001); Lebanon: −3.14% AAPC (p<0.001); Puerto Rico: −3.35% AAPC (p<0.001)). The highest IHD burden in T2DM-Dominant Regions was observed in Cameroon at 10,078.12 per 100,000 in 2021.

IHD-Dominant Regions, while characterized by lower T2DM burden relative to IHD, still demonstrated substantial disease load across 46 countries and territories (**Supplementary Table S3**). Eastern European countries in this category showed varying IHD burden (eg, Poland: 10,278.52 per 100,000 in 2021 [95% UI: 9,117.11–11,193.11]; Slovakia: 16,151.72 per 100,000 [95% UI: 13,671.31–18,479.43]). Nigeria demonstrated the lowest burden within this quadrant (10,178.39 per 100,000 [95% UI: 8161.38 – 12,389.93]), while Ukraine showed the highest burden (33,373.97 per 100,000 [95% UI: 25,405.4 to 41,834.62]). Georgia achieved the most significant improvement (AAPC: -3.4677, p<0.001), while Montenegro showed the greatest deterioration (AAPC: 0.8611, p=0.479). Most countries showed declining IHD trends with negative AAPC values.

Dual-Burden Regions demonstrated high disease burden for both conditions globally, with substantial variation across 56 countries and territories (**Supplementary Table S4**). Among Pacific Island nations, Fiji exhibited IHD burden of 24,758.17 per 100,000 in 2021 (95% UI: 19,028.79–31,212.44), American Samoa at 16,234.35 per 100,000 (95% UI: 13,298.30–19,661.85), and Marshall Islands at 25,107.72 per 100,000 (95% UI: 19,093.64–32,237.25). Gulf states demonstrated substantial burden, including Bahrain at 13,966.71 per 100,000 (95% UI: 11,553.18–16,596.17) and Qatar at 11,003.65 per 100,000 (95% UI: 8,262.37–14,248.67). Kuwait exhibited the lowest IHD burden in this category (10,291.18 per 100,000 [95% UI: 8324.35 - 12533.16]), while Nauru demonstrated the highest (39,483.92 per 100,000 [95% UI: 26,455.3–38,550.96]). The most substantial improvement was seen in Qatar (AAPC: -3.6960, p<0.001), while Cabo Verde experienced the greatest deterioration (AAPC: 1.8900, p<0.001).

### T2DM burden patterns across four regions

For T2DM burden analysis in Low-Burden Regions (**Supplementary Table S5**), countries showed relatively modest disease impact. Sweden showed increase of 0.51% AAPC (p<0.001) and Iceland showed increase of 1.26% AAPC (p<0.001), indicating rising T2DM burden even in high-income settings. For T2DM burden in T2DM-Dominant Regions (**Supplementary Table S6**), Cook Islands exhibited the highest burden at 16,073.11 per 100,000 in 2021, followed by Mauritius at 14,979.32 per 100,000. However, T2DM trends were mixed, with several countries showing concerning increases (Guatemala: 3.81% AAPC [p<0.001]; El Salvador: 2.34% AAPC [p<0.001]; Lesotho: 2.88% AAPC [p<0.001]).

For T2DM in IHD-Dominant Regions, there was considerable variation (**Supplementary Table S7**), with Nepal showing the highest burden at 4,960.94 per 100,000, followed by Sierra Leone at 4,895.92 per 100,000. Several countries demonstrated significant T2DM increases (Uzbekistan: 3.44% AAPC [p<0.001]; Russian Federation: 2.90% AAPC [p<0.001]. In contrast, Belarus showed the lowest T2DM burden at 1,228.79 per 100,000. For T2DM in Dual-Burden Regions, Pacific Island nations dominated the highest burden rankings (**Supplementary Table S8**), with Fiji leading at 32,197.24 per 100,000, followed by Kiribati (22,304.06), Marshall Islands (22,122.46), Nauru (19,303.2), and Bahrain (17,025.47). Several countries experienced significant annual increases in T2DM burden, including Afghanistan (2.27%, p<0.001), Egypt (2.86%, p<0.001), and Morocco (2.89%, p<0.001).

### Risk factor attribution patterns across burden regions

Regarding PAF of risk factors for IHD and T2DM burden, high systolic blood pressure contributed most to IHD burden globally, accounting for 49.79%, while high fasting plasma glucose dominated T2DM burden at 99.99% (**Table 1**). For regional variations, PAFs in Low-Burden Regions were 51.52% for high systolic blood pressure in IHD and 99.99% for high fasting plasma glucose in T2DM. In T2DM-Dominant Regions, high systolic blood pressure accounted for 54.62% of IHD burden while high fasting plasma glucose accounted for 99.99% of T2DM burden. In IHD-Dominant Regions, high systolic blood pressure accounted for 54.89% of IHD burden while high fasting plasma glucose accounted for 99.98% of T2DM burden. In Dual-Burden Regions, high systolic blood pressure accounted for 54.49% of IHD burden while high fasting plasma glucose accounted for 99.98% of T2DM burden. Among environmental factors, household air pollution showed the steepest regional gradient, ranging from 4.58% in Low-Burden Regions to 14.43% in Dual-Burden Regions for IHD, and from 2.19% to 8.44% for T2DM. High body-mass index also demonstrated substantial regional variation, contributing 51.89% to T2DM burden globally, with regional values ranging from 40.61% in IHD-Dominant Regions to 51.36% in Low-Burden Regions.

**Table 1:** Population attributable fractions for IHD and T2MD of various risk factors in 2021.

Risk factors	Global		Low-burden regions		T2DM-dominant regions		IHD-dominant regions		Dual-burden regions	
	IHD	T2DM	IHD	T2DM	IHD	T2DM	IHD	T2DM	IHD	T2DM
Household air pollution from solid fuels	9.52	5.26	4.58	2.19	13.15	6.49	11.27	7.05	14.43	8.44
Ambient particulate matter pollution	19.34	11.81	21.18	12.65	13.64	10.88	18.79	11.91	22.28	11.2
Smoking	18.48	7.16	17.76	8.36	10.92	4.49	14.25	5.9	13.59	5.87
Secondhand smoke	5.13	4.87	5.48	4.61	2.49	3.66	4.03	5.03	4.77	5.32
High fasting plasma glucose	13.72	99.99	15.05	99.99	16.32	99.99	15.54	99.98	18.19	99.98
High body-mass index	12.55	51.89	10.83	51.36	11.96	50.7	10.25	40.61	13.41	49.11
Low physical activity	2.11	7.38	2.55	9.07	1.35	7.15	2.83	10	2.08	8.18
Diet low in fruits	8.38	4.48	3.59	2.22	4.73	3.51	9.03	7.16	4.75	3.8
Diet high in red meat	2.32	5.04	3.63	8.4	1.74	4.85	0.92	1.82	0.37	2.57
Diet low in whole grains	14.3	6.73	11.53	6.65	8.19	3.15	12.53	6.96	13.64	8.91
Diet high in sugar-sweetened beverages	0.11	4.01	0.07	4.92	0.08	4.15	0.06	2.5	0.05	2.1
Diet low in vegetables	3.22	0.79	1.47	0.33	3.94	1.27	3.04	0.63	3.49	2.28
Diet high in processed meat	1	8.07	1.39	11.42	0.12	5.22	0.61	5.51	0.17	6.2
High temperature	1.38	1.24	0.61	0.45	0.64	1.05	1.52	2.05	2.93	2.94
Low temperature	5.31	1.83	6.38	2.12	3.68	2.27	4.85	1.98	4.95	2.07
Lead exposure	6.26	NA	6.4	NA	6.94	NA	7.2	NA	8.57	NA
High systolic blood pressure	49.79	NA	51.52	NA	54.62	NA	54.89	NA	54.49	NA
High LDL cholesterol	35.12	NA	29.04	NA	29.06	NA	29.25	NA	29.63	NA
Kidney dysfunction	14.01	NA	13.86	NA	16.22	NA	16.13	NA	15.94	NA
Alcohol use	-1.76	1.77	-2.01	2.99	NA	NA	NA	NA	-0.73	0.91
Diet low in seafood omega-3 fatty acids	8.18	NA	4.59	NA	6.78	NA	8.06	NA	7.78	NA
Diet high in trans fatty acids	1.31	NA	NA	NA	NA	NA	NA	NA	NA	NA
Diet low in legumes	4.83	NA	3.39	NA	3.24	NA	4.98	NA	3.71	NA
Diet low in polyunsaturated fatty acids	9.36	NA	6.85	NA	8.02	NA	8.94	NA	8.61	NA
Diet low in nuts and seeds	8.58	NA	4.92	NA	4.56	NA	8.7	NA	6.77	NA
Diet low in fiber	6.12	1.03	3.64	NA	4.27	NA	5.07	NA	5.79	NA
Diet high in sodium	7.39	NA	10.78	NA	6.76	NA	6.13	NA	5.01	NA

IHD, ischemic heart disease; T2MD, type 2 diabetes mellitus; NA, not applicable.

**Figure 2:**
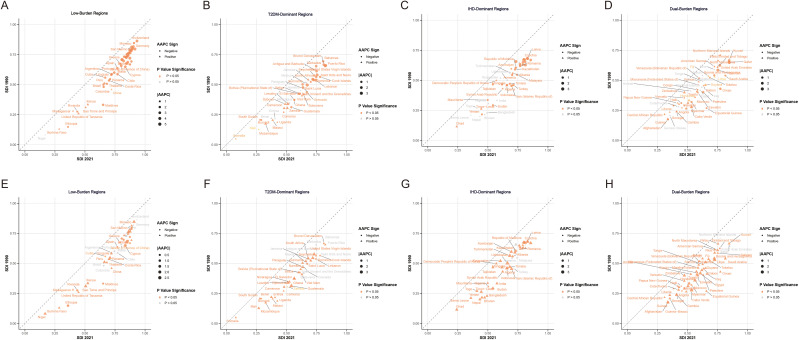
Average annual percentage change in age-standardized disability-adjusted life years rate of IHD **(A–D)** and T2MD **(E–H)** by four characteristic regions from 1990 to 2021. IHD, ischemic heart disease; T2MD, type 2 diabetes mellitus.

**Figure 3:**
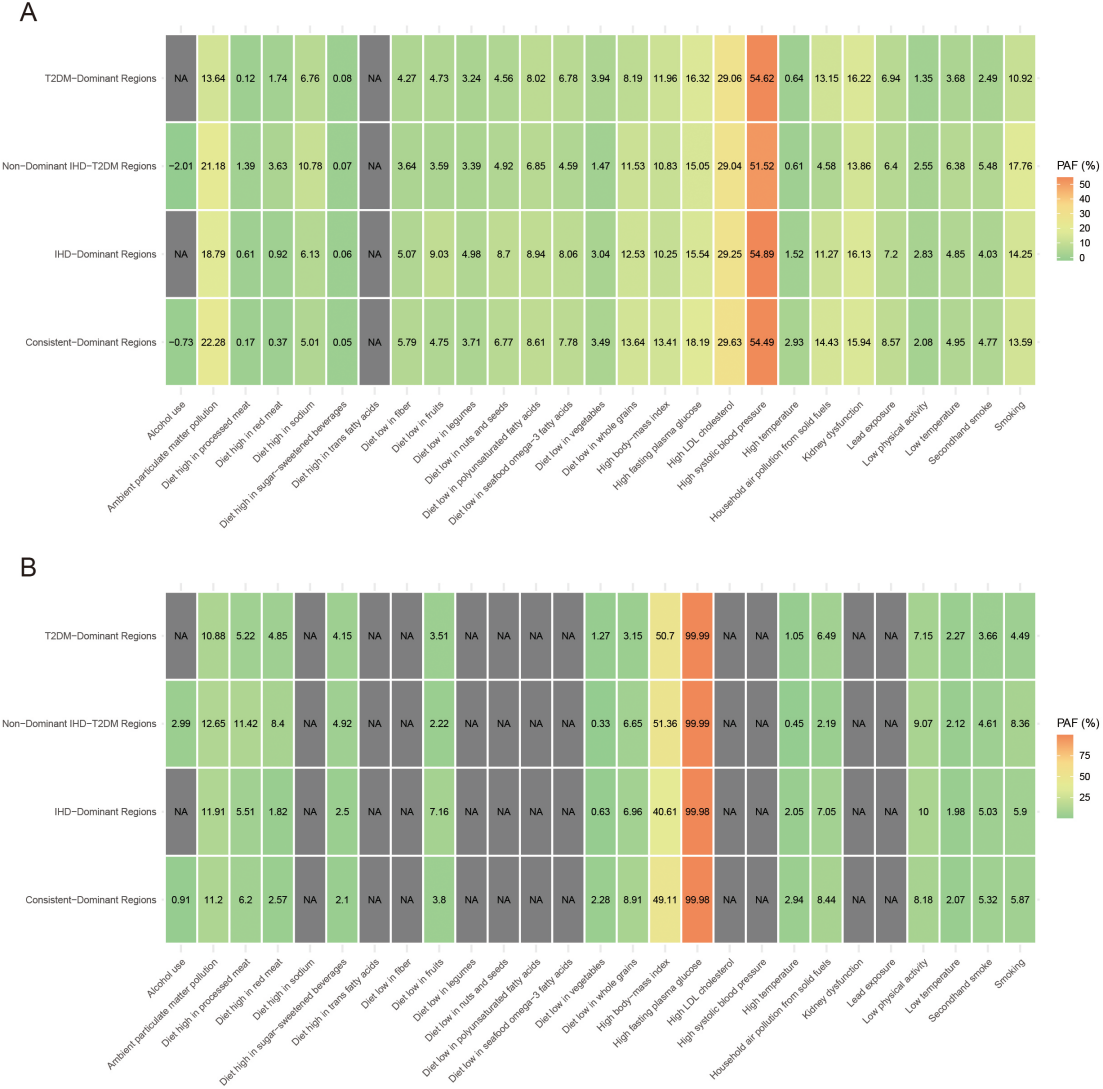
Proportion of the age-standardized disability-adjusted life years rate of IHD **(A)** and T2MD **(B)** among people aged 55 years and older attributable to various risk factors by four characteristic regions in 2021. IHD, ischemic heart disease; T2MD, type 2 diabetes mellitus.

Temporal trends from 1990 to 2021 demonstrated heterogeneous patterns across regional classifications (**Figure 2**). Most dual-burden regions experienced simultaneous social-demographic index (SDI) improvements from 1990 to 2021, yet showed divergent disease trajectories, indicating complex relationships between socioeconomic development and disease outcomes. For the spatial distribution characteristics of major risk factors across different regional classifications, distinct patterns emerged for both IHD and T2DM (**Figure 3**). For IHD risk factors (**Figure 3A**), high systolic blood pressure showed the highest population attributable fraction (PAF) values across all regions, with consistently high contributions across T2DM-Dominant (54.62%), IHD-Dominant (54.89%), and Dual-Burden Regions (54.49%). For T2DM risk factors (**Figure 3B**), high fasting plasma glucose dominated across all regional classifications with PAF values approaching 99.99%. Environmental factors such as household air pollution demonstrated clear regional gradients, with steepest increases from Low-Burden to Dual-Burden Regions.

### Machine learning-based regional comparison and risk factor prioritizations

**Figure 4:**
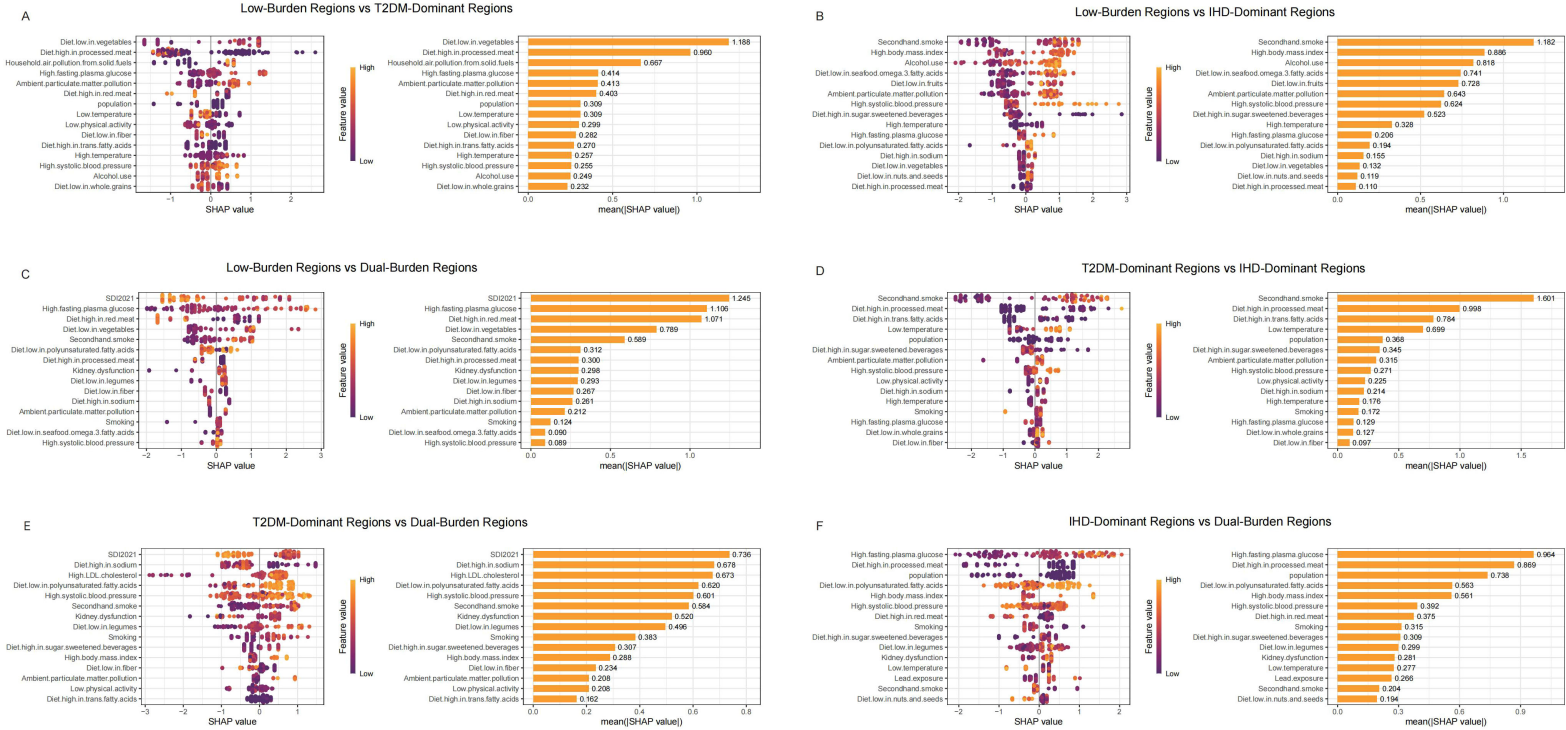
SHAP summary plot of IHD in characteristic regions, Low-Burden Regions vs T2DM-Dominant Regions **(A)**; Low-Burden Regions vs IHD-Dominant Regions **(B)**; Low-Burden Regions vs Dual-Burden Regions **(C)**; T2DM-Dominant Regions vs lHD-Dominant Regions **(D)**; T2DM-Dominant Regions vs Dual-Burden Regions **(E)**; IHD-Dominant Regions vs Dual-Burden Regions **(F)**. Plots display variables ranked by importance in terms of risk factor exposure from top to bottom. The SHAP value on the horizontal axis represents the influence of the factor on the outcome, with positive values indicating promotion and negative values indicating inhibition. The larger the absolute value of SHAP, the greater its impact on population attributable fractions for IHD. IHD, ischaemic heart disease; LDL, low-density lipoprotein; SHAP, Shapley additive explanations.

**Figure 5:**
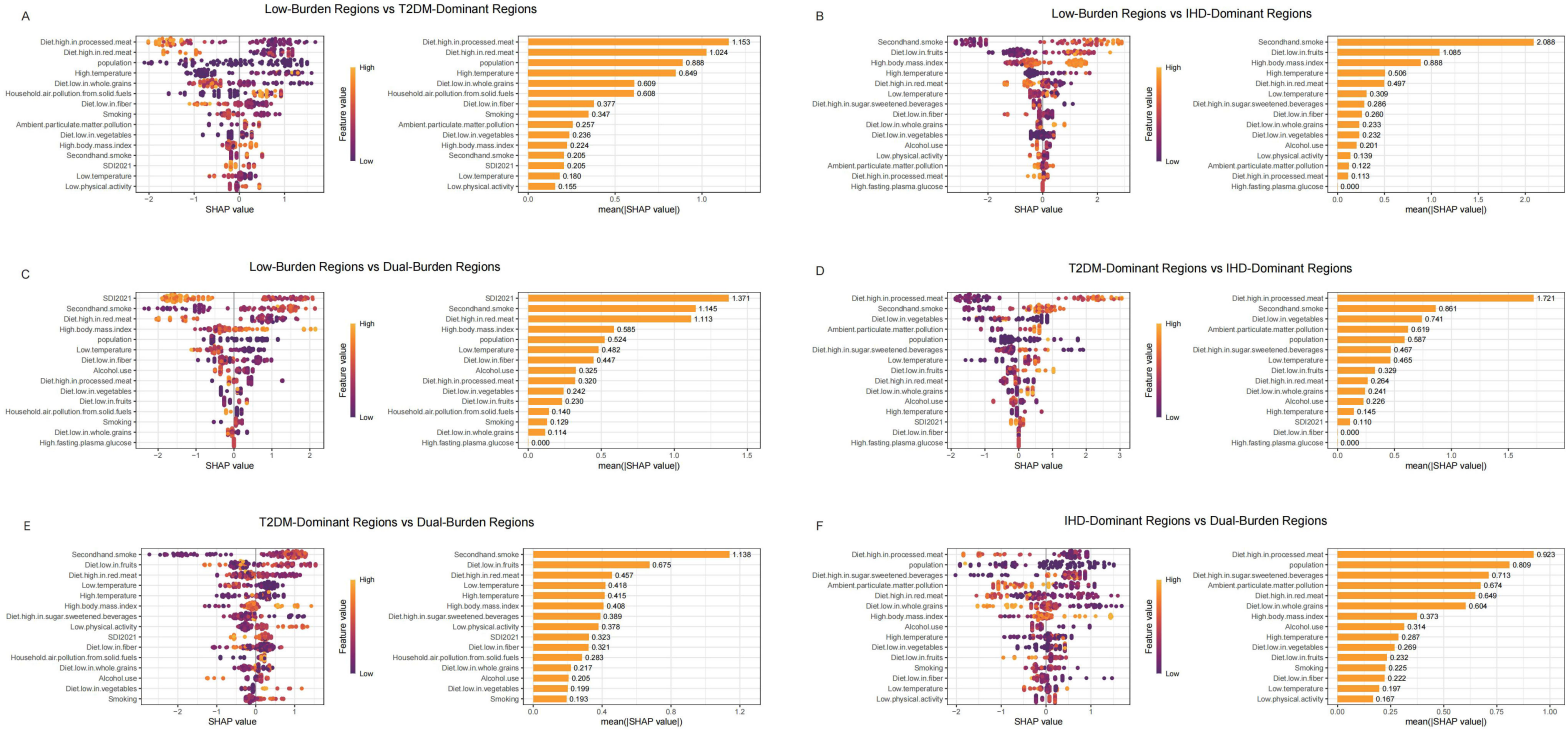
SHAP summary plot of T2MD in characteristic regions, Low-Burden Regions vs T2DM-Dominant Regions **(A)**; Low-Burden Regions vs IHD-Dominant Regions **(B)**; Low-Burden Regions vs Dual-Burden Regions **(C)**; T2DM-Dominant Regions vs lHD-Dominant Regions **(D)**; T2DM-Dominant Regions vs Dual-Burden Regions **(E)**; IHD-Dominant Regions vs Dual-Burden Regions **(F)**. Plots display variables ranked by importance in terms of risk factor exposure from top to bottom. The SHAP value on the horizontal axis represents the influence of the factor on the outcome, with positive values indicating promotion and negative values indicating inhibition. The larger the absolute value of SHAP, the greater its impact on population attributable fractions for T2MD. T2MD, type 2 diabetes mellitus; SHAP, Shapley additive explanations.

We used SHAP analysis to investigate risk factors distinguishing regional classifications across both diseases (**Figures 4**, **5**). Our SHAP analysis revealed distinct risk factor profiles that differentiate the disease burden regions, with the most critical insights emerging from the comparison between Low-Burden and Dual-Burden Regions. For IHD, the SDI in 2021 was the paramount factor distinguishing these regions (mean |SHAP| = 1.245), indicating that fundamental socioeconomic development levels underpin the co-occurrence of high IHD and T2DM burden. Beyond SDI, a constellation of dietary risks were highly influential, including high fasting plasma glucose (mean |SHAP| = 1.106), diet high in red meat (1.071), and diet low in vegetables (0.789) (**Figure 4C**). This suggests that dual-burden regions are characterized not only by lower socioeconomic development, but also by a synergistic risk profile where adverse metabolic conditions and suboptimal dietary patterns converge. For T2DM, a similar pattern emerged, with SDI reconfirmed as the primary differentiator (mean |SHAP| = 1.317). This was closely followed by the substantial influence of secondhand smoke (mean |SHAP| = 1.145). The model also identified a cluster of dietary risks, with high red meat intake (1.113) and high body-mass index (0.585) being prominent contributors highlighting a pervasive nutritional transition that characterizes the T2DM component of the dual burden (**Figure 5C**).

For IHD regional distinctions (**Figure 4**), diet low in vegetables emerged as the primary differentiator between Low-Burden and T2DM-Dominant Regions (mean |SHAP| = 1.188), followed by Diet high in processed meat (0.960) and household air pollution from solid fuels (0.667) (**Figure 4A**). When comparing Low-Burden and IHD-Dominant Regions, secondhand smoke showed exceptional importance (mean |SHAP| = 1.182), along with high body mass index (0.886) (**Figure 4B**). Among T2DM-Dominant and IHD-Dominant Regions, secondhand smoke demonstrated notable differentiation (mean |SHAP| = 1.601) (**Figure 4D**). When comparing T2DM-Dominant with Dual-Burden Regions, SDI 2021 emerged as the leading differentiator (mean |SHAP| = 0.736), followed by Diet high in sodium (0.678) (**Figure 4E**). Similarly, for IHD-Dominant versus Dual-Burden Regions, high fasting plasma glucose showed the highest importance (mean |SHAP| = 0.964), with diet high in processed meat also contributing significantly (0.869) (**Figure 4F**).

For T2DM regional distinctions (**Figure 5**), diet high in processed meat showed the strongest differentiation between Low-Burden and T2DM-Dominant Regions (mean |SHAP| = 1.153) (**Figure 5A**). When distinguishing Low-Burden from IHD-Dominant Regions, secondhand smoke demonstrated exceptional importance (mean |SHAP| = 2.088) (**Figure 5B**). When comparing T2DM-Dominant with Dual-Burden Regions, secondhand smoke emerged as a leading differentiator (mean |SHAP| = 1.138) (**Figure 5E**). Among other dominant region comparisons, diet high in processed meat consistently showed strong discriminatory power, with particularly high importance in T2DM-Dominant versus IHD-Dominant Regions (1.721) and substantial contribution in IHD-Dominant versus Dual-Burden Regions (0.923) (**Figures 5D, F**).

## Discussion

In this study, we proposed a global co-occurrence pattern of IHD and T2DM from a spatial perspective based on country-level DALY rates. We divided 204 countries and territories into four different types of co-occurrence regions: Low-Burden Regions (27.45%), T2DM-Dominant Regions (22.55%), IHD-Dominant Regions (22.55%), and Dual-Burden Regions (27.45%). This classification system emerges at a critical juncture when global health systems face unprecedented challenges from COVID-19, climate change, and economic uncertainties that disproportionately affect populations with chronic diseases (25–27). We then functionally distinguished and quantitatively evaluated the risk factors in every pattern using both traditional epidemiological approaches and machine learning techniques. The results showed that the spatial distribution of the four co-occurrence patterns overlapped with exposure to environmental, dietary, and behavioral risk factors. Overall, high systolic blood pressure contributed 49.79% to global IHD burden, while high body-mass index accounted for 51.89% of T2DM burden, with specific combinations and variations of these risk factors greatly influencing the global disparities and patterns of IHD and T2DM burden. These findings provide crucial evidence for achieving the UN Sustainable Development Goal 3.4, which aims to reduce premature mortality from non-communicable diseases by one-third by 2030 (28).

IHD and T2DM share common risk factors and are related to similar pathophysiological mechanisms including endothelial dysfunction, chronic inflammation, and accelerated atherosclerosis, and it is common to encounter patients with both conditions in clinical practice (29–31). Our results show distinct geographical distribution patterns, with dual-burden regions predominantly concentrated in Pacific Island nations, Gulf states, and parts of Central Asia and Africa. Among Pacific Island nations, Fiji, Marshall Islands, and Nauru demonstrated exceptionally high burden levels for both diseases, with Nauru showing the most extreme burden globally. Gulf states also exhibited substantial dual disease burden, though Qatar demonstrated significant improvement trends while maintaining dual-burden status. These findings extend previous research that focused on single diseases or limited geographic regions, revealing the complex global landscape of dual disease burden.

In a global context, our findings suggest that country-level risk factor co-occurrence rates and exposure rates were highly integrated with the distribution of socioeconomic development status, consistent with previous reports showing close relationships between chronic disease burden and economic status (32–34). Our SHAP analysis revealed that SDI served as the primary factor distinguishing dual-burden regions from low-burden regions, indicating fundamental relationships between development patterns and disease co-occurrence. This finding has profound implications for current global economic recovery efforts post-COVID-19, as it suggests that traditional development approaches may paradoxically increase vulnerability to dual disease burdens (35, 36). Countries in dual-burden regions were predominantly small island developing states and middle-income countries facing rapid epidemiological transitions—populations identified as particularly vulnerable to climate change impacts and rising sea levels (36–38). The concentration of dual-burden regions in Pacific Island nations directly aligns with current international climate justice discussions and the urgency of addressing health impacts in climate-vulnerable regions (38–40). Conversely, countries with the lowest burden levels were concentrated among high-income nations with well-established healthcare systems, though some Nordic countries showed concerning upward trends in diabetes burden, highlighting that even wealthy nations face emerging chronic disease challenges amid changing global conditions (41, 42). Furthermore, while the pathophysiological link between hyperglycemia and type 2 diabetes is unequivocal, underscores a biological definition rather than a practical interventional target. For actionable public health strategies, our focus must shift to the modifiable risk factors that drive the development of hyperglycemia and subsequent dual disease burden, such as household air pollution and dietary patterns, which represent the most leverageable points for prevention.

Our study revealed household air pollution as a significant environmental risk factor showing steep regional gradients, with substantially higher contributions in dual-burden regions compared to low-burden regions for both diseases. This finding has immediate relevance for global climate action and energy transition policies, as household air pollution disproportionately affects regions that contribute least to global carbon emissions but suffer most from environmental health consequences (43–46). The gradient pattern observed directly supports arguments for climate justice and health co-benefits approaches in international climate negotiations, including the COP meetings and Paris Agreement implementation strategies (47). Previous studies have established that environmental exposures contribute significantly to cardiovascular and metabolic disease burden, especially in low-income countries facing energy poverty, a challenge exacerbated by recent global energy crises and geopolitical conflicts affecting energy supply chains (48). Because clean energy access is regarded as an effective intervention to reduce environment-related disease burden, advocating for universal access to clean energy aligns with multiple intergovernmental priorities, addressing simultaneous objectives of promoting global health, environmental sustainability, and energy security in an increasingly unstable geopolitical environment (49).

Our study provides robust evidence supporting major effects of dietary habits on both IHD and T2DM, with critical implications for global food security and sustainable food systems amid current global crises (50). Our SHAP analysis identified dietary factors as key differentiators between regional burden classifications, with vegetable consumption deficiency and processed meat consumption patterns showing strong discriminatory power across multiple regional comparisons. These findings emerge at a time when global food systems face unprecedented disruptions from climate change, conflicts affecting major grain-producing regions, particularly the Russia-Ukraine conflict, and supply chain instabilities that disproportionately affect vulnerable populations’ access to healthy foods (51, 52). Countries in IHD-dominant regions showed higher exposure to dietary risk factors, while T2DM-dominant regions were characterized by specific nutritional deficiencies, patterns that may be exacerbated by current global food price inflation and accessibility challenges (53, 54). In addition, high fasting blood glucose highlights the crucial role of abnormal blood glucose in driving the burden in these regions as a key pathophysiological link between the two diseases. This is related to a diet pattern characterized by excessive consumption of red meat, with increased intake of saturated fat and heme iron, and low vegetable intake, which means a lack of protective fiber, antioxidants and micronutrients. It indicates a nutritional transformation into a diet that simultaneously promotes insulin resistance, dyslipidemia and vascular inflammation (50, 53). In dual-burden regions, the complex interaction between underlying socioeconomic disadvantages and a series of modifiable metabolic and dietary risks has created an environment where both IHD and T2MD are prevalent. The prominence of dietary factors in our analysis directly supports calls for transformation of global food systems toward sustainability and health, aligning with the UN Food Systems Summit commitments and the EAT-Lancet Commission recommendations for planetary health diets (55). Policy makers should prioritize the development and implementation of accessible and culturally appropriate dietary guidelines, particularly in low-income and middle-income countries where such guidelines are often lacking and where populations face increasing food insecurity. Global collaboration, such as partnerships facilitated by WHO and the new Food and Agriculture Organization initiatives, could play a crucial role in disseminating effective dietary interventions while addressing concurrent challenges of food security, climate adaptation, and economic recovery (56).

Compared with existing research, our study made several breakthroughs that are particularly valuable. First, we adopted a spatial perspective to systematically examine the co-occurrence of IHD and T2DM on a global scale, successfully identifying four distinct regional burden profiles that reflect complex interactions of socioeconomic, environmental, and behavioral factors, essential intelligence for navigating the “polycrisis” era of intersecting health, climate, and economic challenges (57–59). Second, we employed advanced machine learning techniques, specifically SHAP analysis, to quantitatively evaluate risk factor importance in distinguishing regional classifications, providing actionable insights for targeted interventions that can maximize impact with limited resources, a critical consideration given current global economic constraints and competing health priorities. This approach differs from previous research that typically examined these diseases separately or relied on traditional statistical methods, and offers sophisticated analytical tools needed for evidence-based decision-making in complex, rapidly-changing global environments. Our findings provide essential baseline data for understanding how global disruptions may alter disease burden trajectories. Lastly, our quadrant classification system provides a practical framework for international resource allocation and policy development at a time when global health financing faces unprecedented pressures and demands for accountability and efficiency have never been higher.

We acknowledge several limitations to this analysis. First, the cross-sectional nature of burden classification may not capture countries undergoing rapid epidemiological transitions or recent disruptions from global events (21). Second, the GBD methodology, while comprehensive, relies on modeling approaches that may not fully capture local variations in disease patterns or healthcare access quality (60). Third, our analysis focused on adults aged ≥55 years, which may not reflect disease burden patterns in younger populations or capture early intervention opportunities. Fourth, temporal trends analysis was limited to the study period and may not reflect recent changes from ongoing conflicts or climate-related disruptions affecting vulnerable regions. Finally, while our SHAP analysis identified key risk factor differentiators, the complex interactions between multiple risk factors and their cumulative effects over time require further investigation through longitudinal studies.

## Conclusion

In conclusion, research on cardiovascular and metabolic diseases should adopt an integrated approach, addressing both single diseases and co-occurrence patterns while acknowledging their unique regional characteristics. The identification of dual-burden regions comprising over a quarter of global countries highlights the urgent need for coordinated international responses that go beyond traditional disease-specific approaches. Our findings provide critical evidence for implementing targeted prevention strategies and achieving Universal Health Coverage goals in an era of global health challenges. Equally important is the alignment of global health strategies with local contexts and capabilities, recognizing that effective interventions must address underlying vulnerabilities that predispose populations to both environmental and disease-related threats. Only through this comprehensive approach, supported by evidence-based risk factor prioritization and innovative analytical methods, can IHD and T2DM be effectively prevented and managed, ultimately enhancing global health security and contributing to sustainable development goals.

## Data Availability

The datasets presented in this study can be found in online repositories. The names of the repository/repositories and accession number(s) can be found in the article/**Supplementary Material**.
