# Supplementary material for: Co-occurrence patterns and related risk factors of ischemic heart disease and type 2 diabetes in burden of disability-adjusted life years among people aged 55 years and older across 203 countries and territories
Source: Front Endocrinol (Lausanne). 2025 Nov 27;16:1693166. doi: 10.3389/fendo.2025.1693166 (PMC12695565; doi:10.3389/fendo.2025.1693166)
Supplement: Supplementary file 1 [file DataSheet1.docx]

**Supplemental Methods**

**1. GBD data processing and modeling process.**

GBD 2021 compiled data sources through data identification and extraction. By identifying from systematic reviews, government and international organisation websites, published reports, primary data sources and contributions of datasets by GBD collaborators, the GBD study combined a variety of data sources including censuses, household surveys, civil registration and vital statistics, disease registries, health service use, air pollution monitors, satellite imaging, disease notifications and other sources, among which each of them was given a unique identifier and included in the Global Health Data Exchange (GHDx) (1). The systematic review in GBD 2021 mainly included three approaches: (1) electronic searches in databases, (2) grey literature searches and (3) consultation from experts. Garbage code redistribution and noise reduction data, together with small sample size were excluded. Data sources used in estimating the burden of ischemic heart disease and type 2 diabetes mellitus across different countries and territories worldwide can be found in the GBD 2021 data input source tool (<http://ghdx.healthdata.org/gbd-2021/datainput-sources>) (1, 2).

GBD 2021 corrected bias and adjusted data for further modelling process. The collected data were modelled by spatiotemporal Gaussian process regression to allow for smoothing over age, time and location in locations lacking complete datasets. The Meta-Regression with Bayesian priors, Regularisation and Trimming (MR-BRT) programme was adopted to adjust data bias for alternative case definitions and study methods. There are three types of age splitting and sex splitting: (1) Estimates were split by age and sex, where possible. Nevertheless, age-­ specific estimates would be calculated based on the reported sex ratio and uncertainty bounds when the incidence data were reported for specific age groups without separating by sex, or by sex for large age intervals; (2) The remaining individual sex estimates were performed with MR-BRT. MR­BRT network meta-analysis estimated the pooled sex ratios and uncertainty bounds, which were then adopted to split combined sex estimates and (3) Researches presenting prevalence estimates for age groups of 25+ years were split into 5-­year age groups using the age pattern generated by DisMod-MR 2.1 (1, 2).

GBD 2021 modelled the epidemiology of ischemic heart disease and type 2 diabetes mellitus using a meta-regression tool based on Bayesian model framework, namely, DisMod-MR 2.1. The Bayesian approach serves as an interpretation of statistical probability, where existing data are used to inform the probability of a given hypothesis. A meta-regression can be regarded as an extension of a meta-analysis whereby data from different sources are pooled into a weighted average adjusting for heterogeneities. DisMod-MR 2.1 applied a negative-binomial model of disease incidence, prevalence, remission and case-fatality rates, and fitted models with a randomised Markov-Chain Monte Carlo algorithm. The steps of modelling process in DisMod-MR 2.1 are as follows: (1) It pooled heterogeneous raw data for each parameter and adjusted data for methodological distinctions. If the data were insufficient to indicate an age-pattern variation, DisMod-MR 2.1 may impose a common age pattern according to assessment of age-specific input data for the disease; (2) It checked data on incidence, prevalence, duration, remission and mortality risk for internal consistency; (2) It simultaneously integrated the input data from all parameters plus to the outputs from previous steps to obtain internally consistent epidemiological estimates, carrying forward uncertainty from primary data sources; (1) Even for countries with little or no primary data source, this model could produce estimates based on information from the available data, and this process allowed for estimates of ischemic heart disease and type 2 diabetes mellitus burden in all countries worldwide (1, 2).

**2. Case definition**

**2.1** Ischemic heart disease (IHD) is a disease that limits the supply of blood to the heart. IHD is typically due to the narrowing of the coronary arteries, usually due to atherosclerosis, which limits blood flow. GBD estimates IHD as the aggregate of discrete sequelae, consisting of myocardial infarction (heart attacks), angina (stable ischemic heart disease manifesting as chest pain), or ischemic cardiomyopathy (heart failure due to IHD). For GBD 2021, we modelled prevalence and incidence of acute myocardial infarction (MI) as well as the prevalence of chronic ischemic heart disease (IHD).

1) Acute myocardial infarction (MI): Definite and possible MI according to the fourth universal definition of myocardial infarction:

a. When there is clinical evidence of myocardial necrosis in a clinical setting consistent

with myocardial ischemic or

b. Detection of a rise and/or fall of cardiac biomarker values and with at least one of the following: i) symptoms of ischemic, ii) new or presumed new ST-segment-T wave

changes or new left bundle branch block, iii) development of pathological Q waves in

the ECG, iv) imaging evidence of new loss of viable myocardium or new regional wall

motion abnormality, or v) identification of an intracoronary thrombus by angiography or autopsy.

c. Sudden (abrupt) unexplained cardiac death, involving cardiac arrest or no evidence of a non-coronary cause of death. The prevalence of MI is considered to last from the onset of the event to 28 days after the event and is divided into an acute phase (0–2 days) and subacute phase (3–28 days). We also included unstable angina when reported separately as specified in the fourth universal definition.
2) Chronic IHD

a. Stable angina: clinically diagnosed stable exertional angina pectoris or definite angina pectoris according to the Rose Angina Questionnaire (RAQ), physician diagnosis, or taking nitrate medication for the relief of chest pain.

b. Asymptomatic ischemic heart disease following myocardial infarction; survival to 28 days following incident MI. The GBD study does not use estimates based on ECG

evidence for prior MI, due to its limited specificity and sensitivity. Reference and alternate definitions of acute myocardial infarction and stable angina are shown in Tables 1a and 1b. ICD codes mapped to acute myocardial infarction and stable angina are listed in Tables 2a and 2b (1, 3).


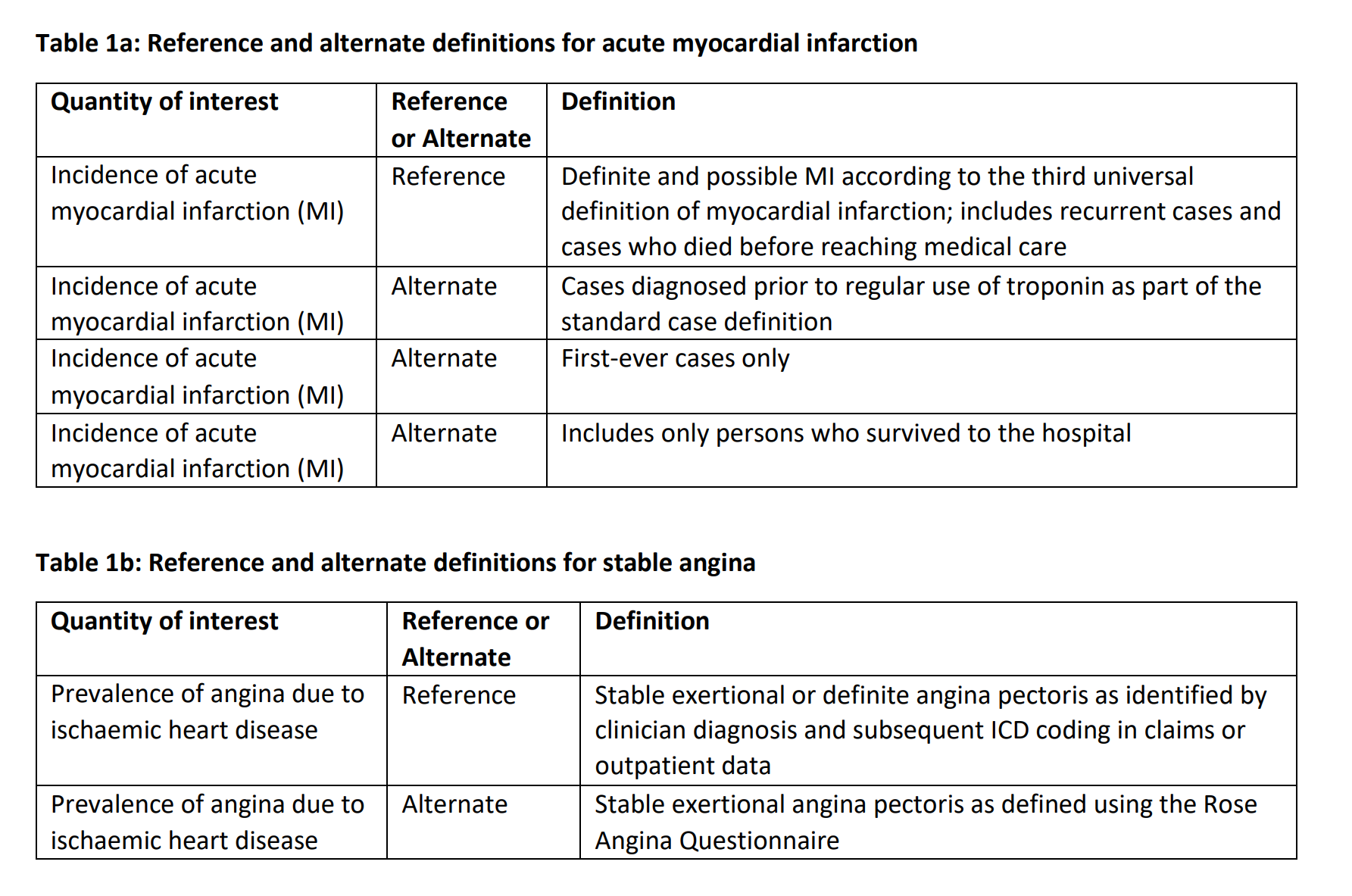


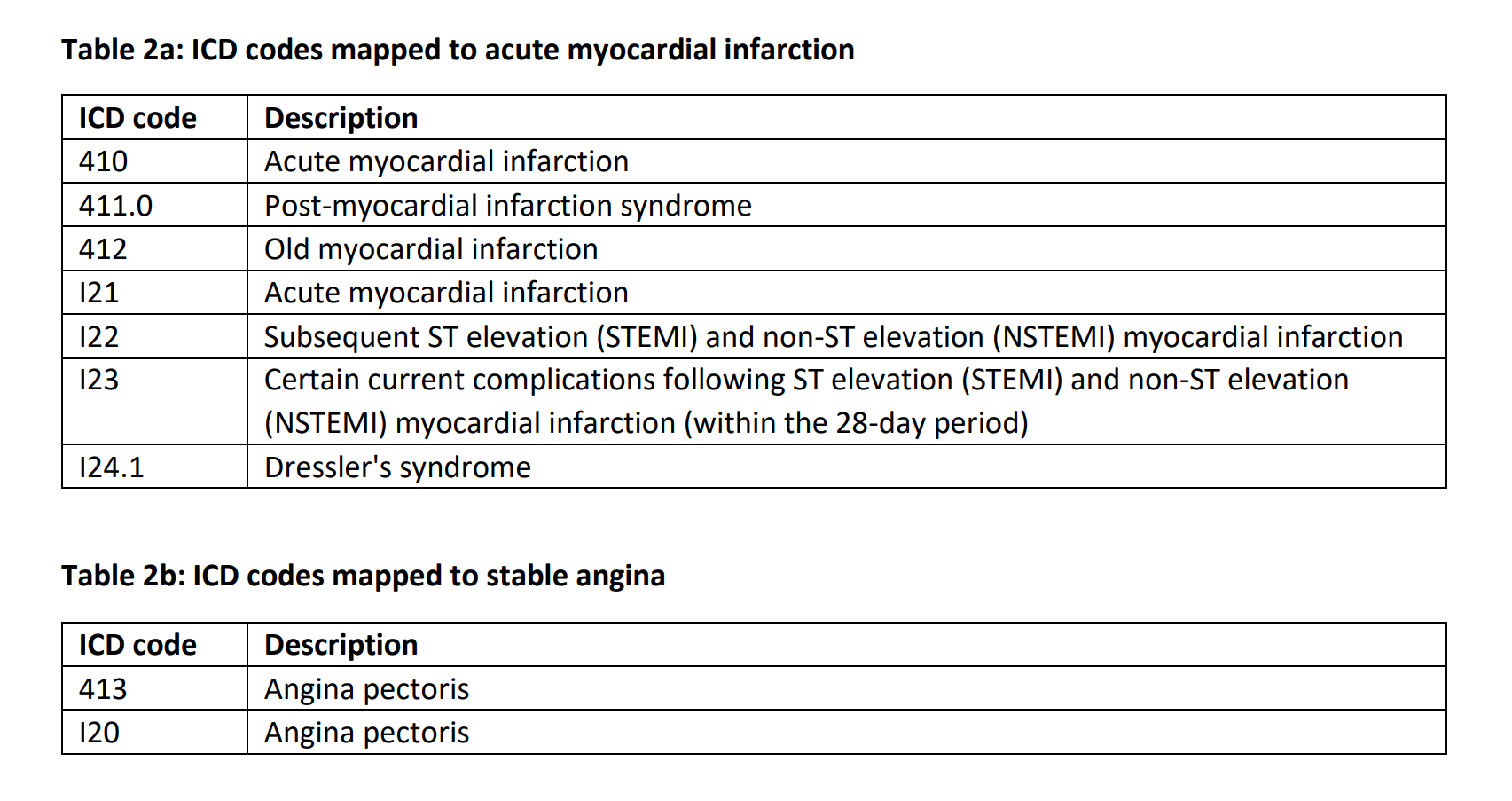


**2.2** For type 2 diabetes mellitus (T2DM), clinical, reference, and alternative case definitions and diagnostic criteria are presented in the table below.

Table : Case definitions for diabetes mellitus


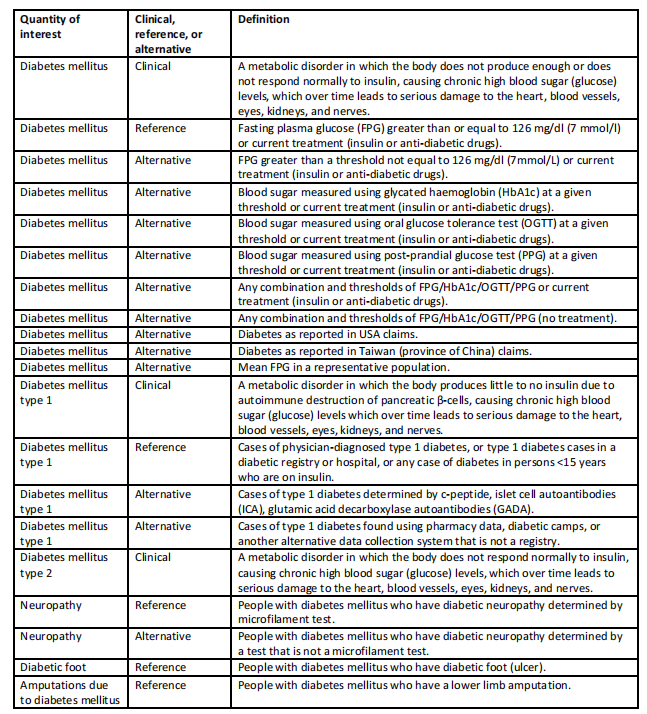


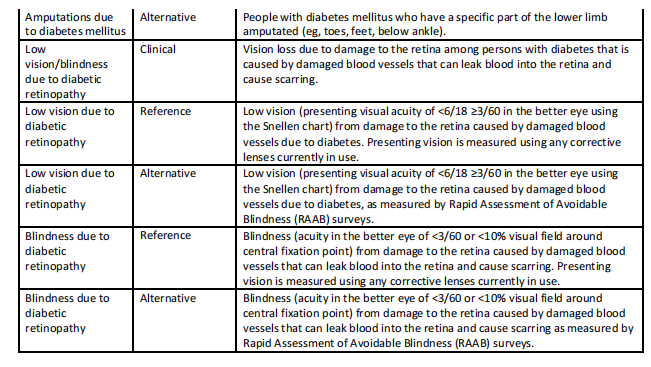


Main exclusion reasons include duplicative studies, not population representative, and self-report of diabetes status.

**3. Risk factor**

Below are the definitions and explanations of each risk factor

| Risk factor | Definition | TMREL |
| --- | --- | --- |
| **Environment risks** |  |  |
| High temperature | High temperature is defined as a daily mean temperature warmer than the TMREL, the temperature with the minimum mortality for all included causes. The population-weighted mean TMREL is 25.6°C, with a range of 21.3–26.6°C. | temparture associated with the lowest mortality for all included causes |
| Low temperature | Low temperature is defined as a daily mean temperature colder than the TMREL, defined as the temperature with the minimum mortality for all included causes. The population-weighted mean TMREL is 25.6°C.. | temparture associated with the lowest mortality for all included causes |
| Ambient particulate matter pollution | Ambient particulate matter pollution is the population-weighted annual average mass concentration of particles with an aerodynamic diameter less than 2.5 micrometres in a cubic metre of air. The TMREL is a uniform distribution between 2.4 and 5.9 µg/m3 . | 2.4–5.9 µg/m^3^ |
| Household air pollution from solid fuels | Household air pollution includes exposure to particulate matter less than 2.5 micrometres in diameter (PM2.5 ) due to the use of solid fuels for cooking, including coal, charcoal, wood, agricultural residue, and animal dung. | 2.4–5.9 µg/m^3^ |
| Lead exposure | There are two types of lead exposure: acute exposure, measured by micrograms of lead per decilitre of blood, and chronic exposure, measured by micrograms of lead per gram of bone. | 0.016 µg/dL |
| **Behavior risk** |  |  |
| Alcohol use | Alcohol consumption in excess of the region-, age-, sex-, and year-specific theoretical minimum risk exposure level (TMREL). Current drinkers are defined as individuals consuming at least one alcoholic beverage in the past year. We estimate the level of alcohol exposure for current drinkers with the reported average grams of pure alcohol consumed per day (g/day) | exposure that minimises your risk of suffering burden from any given cause related to alcohol |
| Smoking | Smoking is defined as current daily or occasional use of any smoked tobacco product. | zero exposure |
| Secondhand smoke | This risk factor refers to any current exposure to secondhand tobacco smoke at home or at work. Only non-daily smokers are considered to be exposed to secondhand smoke. | zero exposure |
| Low physical activity | Low physical activity was measured in total metabolic equivalent (MET)-minutes per week and was defined as objectively measured, average weekly physical activity (at work, home, transport-related, and recreational) of less than 3600–4400 MET-minutes per week. | 3600–4400 METs |
| Diet low in fruit | Diet low in fruit is defined as average daily consumption (in grams per day) of less than 340–350 grams of fruit including fresh, frozen, cooked, canned, or dried fruit, excluding fruit juices and salted or pickled fruits. | 340–350 g/day |
| Diet low in vegetables | Diet low in vegetables is defined as average daily consumption (in grams per day) of less than 306–372 grams of vegetables, including fresh, frozen, cooked, canned, or dried vegetables and excluding legumes and salted or pickled vegetables, juices, nuts, seeds, and starchy vegetables such as potatoes or corn. | 306–372 g/day |
| Diet low in whole grains | Diet low in whole grains is defined as average daily consumption (in grams per day) of less than 160–210 grams of whole grains (bran, germ, and endosperm in their natural proportion) from breakfast cereals, bread, rice, pasta, biscuits, muffins, tortillas, pancakes, and other sources. | 160–210 g/day |
| Diet low in nuts and seeds | Diet low in nuts and seeds is defined as average daily consumption (in grams per day) of less than 19–24 grams of nuts and seeds, including tree nuts and seeds and peanuts. | 19–24 g/day |
| Diet low in fiber | Diet low in fibre is defined as average daily consumption (in grams per day) of less than 22–25 grams of fibre from all sources including fruits, vegetables, grains, legumes, and pulses. | 22–25 g/day |
| Diet low in seafood omega-3 fatty acids | Diet low in seafood omega-3 fatty acids is defined as average daily consumption (in milligrams per day) of less than 470–660 milligrams of eicosapentaenoic acid (EPA) and docosahexaenoic acid (DHA) from seafood sources. | 470–660 mg/day |
| Diet low in polyunsaturated fatty acids (PUFA) | Diet low in omega-6 polyunsaturated fatty acids is defined as average daily consumption (in % daily energy) of less than 9–10% total energy intake from omega-6, specifically linoleic acid, γ-linolenic acid, eicosadienoic acid, dihomo-γ-linolenic acid, and arachidonic acid. | 9–10% of total daily energy |
| Diet low in calcium | Calcium intake is defined as average daily consumption of dietary calcium in grams per day from all sources, including milk, yoghurt, and cheese. The optimal intake for females is defined as 1.1–1.2 grams per day, while the optimal intake for males is defined as 0.72–0.86 grams per day. | 0.72–0.86 g/day (males)  1.06–1.2 g/day (females) |
| Diet low in legumes | Diet low in legumes is defined as average daily consumption (in grams per day) of less than of 100–110 grams of legumes and pulses, including fresh, frozen, cooked, canned, or dried legumes. | 100–110 g/day |
| Diet high in red meat | Diet high in red meat is defined as intake above an average of 0 grams per day (95% UI 0–200) of unprocessed red meat. Unprocessed red meat includes pork and bovine meats such as beef, lamb, and goat, but excludes all processed meats, poultry, fish, and eggs. | 0–200 g/day |
| Diet high in processed meat | Diet high in processed meat is defined as any intake (in grams per day) of meat preserved by smoking, curing, salting, or addition of chemical preservatives. | 0 g/day |
| Diet high in sugar sweetened beverages (SSBs) | Diet high in sugar-sweetened beverages is defined as any intake (in grams per day) of beverages with ≥50 kcal per 226.8-gram serving, including carbonated beverages, sodas, energy drinks, and fruit drinks, but excluding 100% fruit and vegetable juices. | 0 g/day |
| Diet high in trans fatty acids | Diet high in trans fatty acids is defined as intake greater than 0–1.1% daily energy of trans fat from all sources, mainly partially hydrogenated vegetable oils and ruminant products. | 0–1.1 % of total daily energy |
| Diet high in sodium | Diet high in sodium is defined as average 24-hour urinary sodium excretion greater than 1–5 grams per day. | 1–5 g/day |
| **Metabolic risks** |  |  |
| High body-mass index | High BMI for adults (ages 20 and older) is defined as BMI greater than 20–23 kg/m^2^ . High BMI for children (ages 2–19) is defined as being overweight or obese based on International Obesity Task Force standards. | 20–21 kg/m2 |
| High LDL cholesterol | We estimated blood concentration of LDL-c in units of mmol/L. We used a TMREL with a uniform distribution between 0.9 and 1.4 mmol/L. | 0.9–1.4 mmol/L |
| High systolic blood pressure | We estimated brachial SBP in mm Hg. We used a TMREL of SBP ranging from 105 to 115 mm Hg. | 105–115 mmHg |
| Kidney dysfunction | Kidney dysfunction is defined as estimated glomerular filtration rate (eGFR) <60 ml/min/1.73 m^2^ or albumin to creatinine ratio (ACR) ≥30 mg/g. The theoretical minimum risk exposure level value is ACR <30 mg/g and eGFR ≥60 ml/min/1.73 m^2^ . | ACR 30 mg/g or less and eGFR greater than 60ml/min/1.73m^2^ |
| High fasting plasma glucose | High fasting plasma glucose is defined as fasting plasma glucose greater than 4.9–5.3 mmol/L. | 4.88–5.30 mmol/L |

TMREL: theoretical minimum exposure level.

**4. Definition of DALYs**

Disability-adjusted life years (DALYs) are defined as the sum of years lost due to premature death (YLLs) and years lived with disability (YLDs) (1, 2).

**4.1 Calculation methods for DALYs**

To estimate DALYs, GBD 2021 started by estimating cause‐specific mortality and non‐fatal health loss. For each year for which YLDs have been estimated, GBD 2021 computed DALYs by adding YLLs and YLDs for each age-sex-location. Uncertainty in YLLs was assumed to be independent of uncertainty in YLDs. GBD 2021 calculated 1000 draws for DALYs by summing the first draw of the 1000 draws for YLLs and YLDs and then repeating for each subsequent draw. 95% UIs were computed by using the 25th and 975th ordered draw of the DALY uncertainty distribution. GBD 2021 calculated DALYs as the sum of YLLs and YLDs for each cause, location, age group, sex, and year. For more information, please refer to the following figure (1).


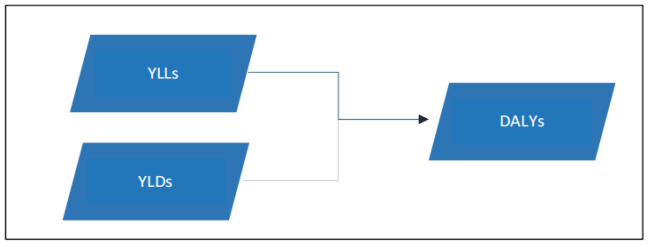


**4.2 YLLs**

The YLL is a metric that is computed by multiplying the number of estimated deaths by the standard life expectancy at age of death. The metric therefore highlights premature deaths by applying a larger weight to deaths that occur in younger age groups. The core equation can be written as follows (1):


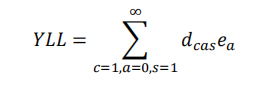


**4.3 YLDs**

YLD was computed by sequela as prevalence multiplied by the DW for the health state associated with that sequela (1).


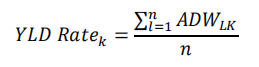


**4.4 Disability Weights (DWs)**

DWs are measured on a scale from 0 to 1; 0 implies a state equivalent to full health, and 1, a state equivalent to death. The formula for the cumulative DW is one minus the multiplicative sum of one minus each DW present (1):


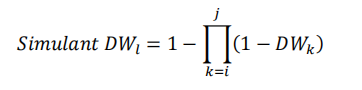


Where:

DW_k_ is the DW for the kth disease sequela that the simulant l has acquired.

Once the simulant DW is computed, the DW attributable to each sequela for the simulant is calculated by using the following formula:


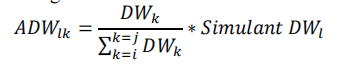


Where:

ADW_lk_ is the attributable DW for disease sequela k in simulant l DW_k_ is the DW for disease sequela k.

Simulant DW_l_ is the DW for simulant l from the combination of all sequelae that they have acquired.

This formula apportions the overall simulant DW to each condition in proportion to the DW of each condition in isolation.

Finally, YLDs per capita in an age-sex-country-year are computed by taking the sum of the attributable DWs for a disease sequela across simulants. The actual number of YLDs from disease sequela k in an age-sex-location-year is then computed as the YLD rate k times the appropriate age-sex-location-year population. GBD 2021 determined the disability weights for each sequela from the GBD disability weight survey (2).

**5. Joinpoint regression analysis**

Time trend analysis is an important component of epidemiological research. Traditional regression models primarily fit and evaluate the overall trend of disease distribution within the study period from a global perspective, failing to capture local variation characteristics. In 1998, Kim et al. first proposed the Joinpoint regression model. The core idea of this model is to establish segmented regression based on the temporal characteristics of disease distribution. By dividing the study time into different intervals through several Joinpoints, the trend in each interval is fitted and optimized, allowing for a more detailed assessment of the specific disease change characteristics within different intervals of the overall time range (3).

The Joinpoint regression model, developed by the Division of Cancer Control and Population Sciences at the National Cancer Institute of the United States, has been widely applied in the field of trend studies on disease incidence and mortality rates.

(I) Model Introduction

The Joinpoint regression model includes two types: the linear model (y = xb) and the logarithmic linear model (ln y = xb). If the dependent variable follows a normal distribution (or approximately normal distribution) and the sample size is large (usually greater than 100), the linear model is preferred. For example, when the dependent variable is continuous variables like height, weight, etc. If the dependent variable follows an exponential distribution or a Poisson distribution, the logarithmic linear model is more suitable. For instance, when the dependent variable represents epidemiological data based on populations such as incidence rates, number of cases, etc. When analyzing trends in the incidence, prevalence, mortality rates, and DALYs rates of thalassemia based on population data, the logarithmic linear model is generally chosen (3).

(II) Modeling Method

The grid search method (GSM) is the default modeling approach used by Joinpoint. GSM divides the study data into a grid, with each grid intersection corresponding to a planned scenario. Then, within the specified intervals, it computes performance metrics for the corresponding equations at each point using a fixed step size to determine the optimal function. In essence, the Joinpoint model uses the GSM to establish all possible segment function Joinpoints (i.e., Joinpoints) and calculates the sum of squares errors (SSE) and mean squared errors (MSE) for each possible scenario. It selects the grid point with the smallest MSE as the Joinpoint for the segment function and fits the equation parameters such as β_0_, β_1_, δ_1_, ..., δ_k_ based on the selected Joinpoints and interval functions (3).

(III) Model Optimization

Monte Carlo permutation test is the default model optimization method in Joinpoint software. Before modeling, it is necessary to set the range of the number of Joinpoints k as k ∈ (MIN，MAX) , where MIN represents the minimum number of Joinpoints, which is usually set to 0; MAX represents the maximum number of Joinpoints. Each permutation test checks the null hypothesis H_0_: the number of Joinpoints is k = k_a_, and the alternative hypothesis H_1_: the number of Joinpoints is k = k_b_. The permutation test starts from k_a_=MIN and k_b_=MAX. If H_0_ is rejected, k is set to k_a_ + 1 for further testing; if H_0_ is not rejected, k is set to k_b_ - 1 for another test, until k_a_ = k_b_, which means k = k_a_ = k_b_ is the preferred number of Joinpoints selected by the permutation test, and the corresponding model is the optimal model (3).

(IV) Index Calculation

Average annual percent change (AAPC) along with their 95% confidence intervals (CI) are the primary outcome indicators of the Joinpoint model. When it comes to assessing the overall average change trend encompassing multiple intervals, the AAPC is required. The parameter calculation method of AAPC involves weighted calculation of the regression coefficients of each interval based on the width w of the segment intervals. Its formula is as follows:


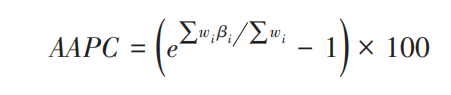


The lower and upper limits of the 100(1-α) % confidence interval are respectively:


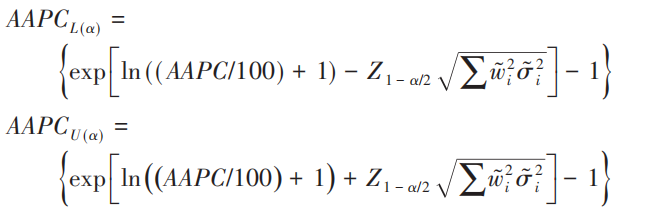


In the above formula, w_i_ represents the width of each segment function interval (i.e., the number of years included in the interval), β_i_ denotes the regression coefficient corresponding to each interval, σ^2^i is the variance of β_i_, and Z_α_ represents the corresponding value of the α percentile in the normal distribution (4,5).

(V) Software Download

To download Joinpoint software, we visited the website of the National Cancer Institute (https://surveillance.cancer.gov/Joinpoint/download), registered, andsubmitted our application information. Software citation: Joinpoint Regression Program, Version 4.9.1.0 - April 2022; Statistical Methodology and Applications Branch, Surveillance Research Program, National Cancer Institute.

**6. XGBoost**

XGBoost, a machine learning algorithm, generates decision trees iteratively to make predictions. With each step, it refines the model by adding new trees aimed at reducing the prediction errors from the previous iteration. The model’s objective function consists of two key components: a loss function, which measures the difference between predicted and actual values, and a regularization term, which limits model complexity to avoid overfitting (6). The objective function can be represented as:

$$\text{ℒ}\left( \text{ϕ} \right)\text{=}\sum_{\text{i}\text{=1}}^{\text{n}} \text{l}\left( {\hat{\text{y}}}_{\text{i}}\text{, }\text{y}_{\text{i}} \right)\text{+ }\sum_{\text{k}\text{=1}}^{\text{K}} \text{Ω}\left( \text{f}_{\text{k}} \right)$$

Where:

$\text{ℒ}\left( \text{ϕ} \right)$ is the overall objective function, combining the loss and regularization components.

$\text{l}\text{(}{\hat{\text{y}}}_{\text{i}}\text{, }\text{y}_{\text{i}}\text{)}$ represents the loss function for each instance, measuring how closely the predicted value ${\hat{\text{y}}}_{\text{i}}$ matches the actual value $\text{y}_{\text{i}}$.

$\text{Ω}\text{(}\text{f}_{\text{k}}\text{)}$ is the regularization term applied to each tree $\text{f}_{\text{k}}$, controlling the complexity of the model to ensure generalization to unseen data.

The regularization term can be expressed as:

$$\text{Ω}\left( \text{f}_{\text{k}} \right)\text{= }\text{γT}\text{+}\frac{\text{1}}{\text{2}}\text{λ}\sum_{\text{j}\text{=1}}^{\text{T}} \text{w}_{\text{j}}^{\text{2}}$$

Where:

$\text{T}$ is the number of leaf nodes in a tree, with more nodes representing a more complex tree.

$\text{w}_{\text{j}}$ denotes the weight assigned to the $\text{j}$-th leaf node, influencing its contribution to the model’s predictions.

$\text{γ}$ penalizes the number of leaf nodes, preventing trees from becoming too large and overfitting.

$\text{λ}$ controls the magnitude of the leaf weights, encouraging smaller weights to make the model more conservative and stable.

**References:**

1. Global incidence, prevalence, years lived with disability (YLDs), disability-adjusted life-years (DALYs), and healthy life expectancy (HALE) for 371 diseases and injuries in 204 countries and territories and 811 subnational locations, 1990-2021: a systematic analysis for the Global Burden of Disease Study 2021. LANCET. [Journal Article]. 2024 2024/5/18;403(10440):2133-61.

2. Global burden of 288 causes of death and life expectancy decomposition in 204 countries and territories and 811 subnational locations, 1990-2021: a systematic analysis for the Global Burden of Disease Study 2021. LANCET. [Journal Article]. 2024 2024/5/18;403(10440):2100-32.

3. Kim HJ, Fay MP, Feuer EJ, Midthune DN. Permutation tests for joinpoint regression with applications to cancer rates. STAT MED. [Journal Article]. 2000 2000/2/15;19(3):335-51.

4. Kim S, Lee S, Choi JI, Cho H. Binary genetic algorithm for optimal joinpoint detection: Application to cancer trend analysis. STAT MED. [Journal Article; Research Support, Non-U.S. Gov't]. 2021 2021/2/10;40(3):799-822.

5. Yang JJ, Trucco EM, Buu A. A hybrid method of the sequential Monte Carlo and the Edgeworth expansion for computation of very small p-values in permutation tests. STAT METHODS MED RES. [Journal Article; Research Support, N.I.H., Extramural]. 2019 2019/10/1;28(10-11):2937-51.

6. Chen T, Guestrin C. Xgboost: A scalable tree boosting system[C]. Proceedings of the 22nd acm sigkdd international conference on knowledge discovery and data mining. 2016: 785-794.

Table S1. In low-burden regions, ASDR of ischemic heart disease among people aged 55 years and older in 1990 and 2021, and change from 1990 to 2021. Abbreviations: ASDR, age-standardized disability-adjusted life years rates; SDI, sociodemographic index; UI, uncertainty interval; AAPC, average annual percent change.

| Location | ASDR, per 100,000 (95% UI) | | AAPC | P value | SDI 1990 | SDI 2021 |
| --- | --- | --- | --- | --- | --- | --- |
|  | 1990 | 2021 |  |  |  |  |
| Andorra | 7622.28 (5388.6 to 10434.89) | 3652.82 (2559.04 to 4919.65) | -2.6774 | <0.001 | 0.76146388 | 0.8694441 |
| Argentina | 14387.18 (13303.12 to 15296.66) | 5688.75 (5116.68 to 6175.48) | -2.8394 | <0.001 | 0.58739728 | 0.7231230 |
| Australia | 16278.17 (14983.94 to 17209.85) | 3697.3 (3206.28 to 4064.12) | -4.8784 | <0.001 | 0.72598252 | 0.8442528 |
| Austria | 15136.17 (13899.33 to 16034.57) | 5903 (5159.49 to 6451.94) | -2.9754 | <0.001 | 0.74985369 | 0.8538370 |
| Belgium | 12335.7 (11167.62 to 13207.71) | 3482.42 (3018.62 to 3845.8) | -3.9926 | <0.001 | 0.73739066 | 0.8536540 |
| Bermuda | 23208.95 (21579.55 to 24709.89) | 6247.07 (5279.24 to 7494.76) | -4.1897 | <0.001 | 0.69645120 | 0.8213654 |
| Brazil | 13002.39 (12159.34 to 13535.92) | 6383.13 (5843.25 to 6750.31) | -2.2084 | <0.001 | 0.50007051 | 0.6530439 |
| Burkina Faso | 8472.91 (6137.57 to 11209.29) | 9693.44 (6811.84 to 13219.65) | 0.5989 | 0.001 | 0.12969562 | 0.2851184 |
| Canada | 14605.36 (13458.99 to 15405.94) | 4517.58 (3988.33 to 4904.87) | -3.6460 | <0.001 | 0.78197786 | 0.8731707 |
| Chile | 10826.79 (9932.55 to 11592.37) | 3707.98 (3313.11 to 4056.76) | -3.3496 | <0.001 | 0.58649510 | 0.7715147 |
| China | 8130.16 (7083.95 to 9229.21) | 9041.47 (7480.93 to 10632.26) | 0.5036 | 0.014 | 0.45866894 | 0.7216298 |
| Colombia | 13925.58 (12962.47 to 14723.79) | 7408.32 (6138.15 to 8751.28) | -2.3700 | <0.001 | 0.48072005 | 0.6554429 |
| Costa Rica | 11655.42 (10681.74 to 12467.33) | 5201.6 (4463.33 to 5861.67) | -2.6460 | <0.001 | 0.53412518 | 0.7003405 |
| Cuba | 19320.99 (18219.46 to 20202.5) | 10026.87 (8673.78 to 11371.13) | -2.2202 | <0.001 | 0.55801907 | 0.6687299 |
| Cyprus | 21541.1 (18223.87 to 25311.05) | 7064.73 (5718.29 to 8467.13) | -3.6220 | <0.001 | 0.64823087 | 0.8356305 |
| Denmark | 19693.99 (18324.37 to 20710.35) | 3557.42 (3100.52 to 3906.87) | -5.4217 | <0.001 | 0.80115466 | 0.8964242 |
| Estonia | 32518.75 (30355.37 to 34129.77) | 8050.49 (6890.92 to 9104.65) | -4.5377 | <0.001 | 0.67496763 | 0.8449178 |
| Ethiopia | 8507.7 (6760.27 to 11057.26) | 5558.38 (4413.28 to 6820.39) | -1.3560 | <0.001 | 0.14803388 | 0.3588233 |
| Finland | 20827.94 (19331.16 to 21917.01) | 6739.16 (5823.34 to 7359.56) | -3.6875 | <0.001 | 0.75622151 | 0.8598314 |
| France | 7531.37 (6850.38 to 8095.46) | 2699.19 (2302.23 to 3025.04) | -3.2556 | <0.001 | 0.73074747 | 0.8383649 |
| Germany | 17437.41 (16025.68 to 18459.56) | 5598.28 (4876.77 to 6125.8) | -3.6058 | <0.001 | 0.81707767 | 0.9029571 |
| Greece | 12761.28 (11744.45 to 13572.44) | 6481.88 (5795.85 to 7022.55) | -2.1799 | <0.001 | 0.67418647 | 0.7918544 |
| Greenland | 19857.09 (16990.9 to 23071.46) | 7742.56 (6250.3 to 9709.47) | -2.8742 | <0.001 | 0.73225825 | 0.8262103 |
| Iceland | 15368.33 (13940.13 to 16419.19) | 4917.06 (4165.96 to 5504.38) | -3.6445 | <0.001 | 0.76421252 | 0.8763617 |
| Ireland | 21852.77 (20411.63 to 22980.9) | 4614.41 (3963.47 to 5096.6) | -4.7033 | <0.001 | 0.71989182 | 0.8737538 |
| Israel | 16380.84 (15123.97 to 17287.94) | 3060.14 (2627.08 to 3385.99) | -5.3154 | <0.001 | 0.70917835 | 0.8090117 |
| Italy | 9882.5 (9090.6 to 10360.66) | 3758.25 (3262.23 to 4056.65) | -3.1502 | <0.001 | 0.70625522 | 0.8057735 |
| Japan | 5685.04 (5162.98 to 5987.89) | 2289.95 (2021.76 to 2452.78) | -3.0048 | <0.001 | 0.79025352 | 0.8712418 |
| Kenya | 4017.46 (2967.99 to 4985.46) | 6011.5 (4573.03 to 7695.26) | 1.3292 | <0.001 | 0.33385029 | 0.5237681 |
| Luxembourg | 14005.17 (12972.14 to 14936.18) | 4086.67 (3559.8 to 4559.91) | -3.8406 | <0.001 | 0.78105161 | 0.8844290 |
| Madagascar | 8214.09 (6224.14 to 10459.88) | 9800.85 (6852.93 to 13253.05) | 0.5094 | <0.001 | 0.27988946 | 0.4002469 |
| Maldives | 16169.42 (12921.06 to 19422.46) | 6871.76 (5384.99 to 8454.01) | -2.9231 | <0.001 | 0.33160154 | 0.6508866 |
| Malta | 19768.43 (18168.99 to 21059.38) | 6308.3 (5411.18 to 7031.76) | -3.7669 | <0.001 | 0.65650458 | 0.8015850 |
| Monaco | 9497.34 (7187.98 to 11805.44) | 4299.1 (3263.54 to 5478.22) | -2.5587 | <0.001 | 0.84549515 | 0.9082628 |
| Netherlands | 13601.59 (12510.61 to 14409.83) | 3232.19 (2805.05 to 3557.91) | -4.6225 | <0.001 | 0.79461212 | 0.8884643 |
| New Zealand | 17829.33 (16528.3 to 18773.97) | 5143.77 (4518.89 to 5595.3) | -3.9742 | <0.001 | 0.75232165 | 0.8494425 |
| Niger | 6886.65 (4456.25 to 10162.66) | 7184.02 (4702.93 to 10292.95) | 0.3186 | 0.072 | 0.08086848 | 0.1680728 |
| Norway | 17029.23 (15987.58 to 17671.87) | 3604.97 (3198.67 to 3872.19) | -5.0495 | <0.001 | 0.79588728 | 0.9161328 |
| Panama | 9902.33 (9013.51 to 10631.65) | 5128.15 (3958.76 to 6142.87) | -1.9724 | <0.001 | 0.54604812 | 0.7088648 |
| Peru | 7247.94 (5861.45 to 8834.66) | 4127.5 (3112.15 to 5422.08) | -1.7149 | <0.001 | 0.51041985 | 0.6620540 |
| Portugal | 10336.91 (9453.97 to 11106.25) | 3363.96 (2923.6 to 3712.32) | -3.5900 | <0.001 | 0.59977776 | 0.7441519 |
| Republic of Korea | 5022.84 (3974.3 to 6172.01) | 2281.7 (1777.96 to 2773.91) | -2.6715 | <0.001 | 0.69232931 | 0.8866753 |
| Rwanda | 9784.2 (6657 to 13969.92) | 6335.42 (4183.15 to 9096.56) | -1.5120 | <0.001 | 0.27509719 | 0.4355887 |
| San Marino | 6789.86 (5451.39 to 8222.32) | 2188.38 (1437.78 to 3108.05) | -4.0248 | <0.001 | 0.81324489 | 0.8880055 |
| Sao Tome and Principe | 7600.53 (5871.16 to 9571.62) | 9871.23 (7665.67 to 12344.9) | 0.9253 | <0.001 | 0.30954285 | 0.5054137 |
| Singapore | 15325.52 (14319.81 to 16230.89) | 4381.42 (3897.49 to 4750.3) | -3.8055 | <0.001 | 0.68640444 | 0.8560978 |
| Slovenia | 12054.78 (10998.43 to 13028.64) | 4233.26 (3574.79 to 4859.34) | -3.3020 | <0.001 | 0.72746393 | 0.8424307 |
| Spain | 8706.56 (7886.88 to 9384.78) | 3225.06 (2803.78 to 3567.63) | -3.1475 | <0.001 | 0.63667317 | 0.7692837 |
| Sweden | 16937.41 (15667.86 to 17755.38) | 4603.99 (3907.85 to 5189.81) | -4.1297 | <0.001 | 0.78553579 | 0.8868803 |
| Switzerland | 12770.71 (11706.83 to 13579.64) | 3337.47 (2813.34 to 3711.63) | -4.2954 | <0.001 | 0.86276684 | 0.9330591 |
| Taiwan (Province of China) | 6765.88 (6112.4 to 7339.16) | 3080.02 (2670.41 to 3433.17) | -2.4526 | <0.001 | 0.66763385 | 0.8747471 |
| Thailand | 7588.81 (5878.96 to 9465.01) | 4267.79 (3119.81 to 5675.07) | -2.0516 | <0.001 | 0.50664486 | 0.6825479 |
| United Kingdom | 19260.22 (18365.98 to 19761.48) | 4750.05 (4359.08 to 4992.18) | -4.5143 | <0.001 | 0.74433413 | 0.8590002 |
| United Republic of Tanzania | 6895.41 (4929.84 to 9298.72) | 9640 (6900.69 to 12938.61) | 1.1023 | <0.001 | 0.25930607 | 0.4465683 |
| United States of America | 16720.89 (15451.01 to 17392.08) | 7189.11 (6492.29 to 7589.24) | -2.7603 | <0.001 | 0.76364769 | 0.8624484 |
| Uruguay | 14252.89 (13127.87 to 15190.29) | 5664.27 (5078.37 to 6172.46) | -3.1321 | <0.001 | 0.58192186 | 0.7192834 |

Table S2. In the type 2 diabetes mellitus-dominant regions, ASDR of ischemic heart disease among people aged 55 years and older in 1990 and 2021, and change from 1990 to 2021. Abbreviations: ASDR, age-standardized disability-adjusted life years rates; SDI, sociodemographic index; UI, uncertainty interval; AAPC, average annual percent change.

| Location | ASDR, per 100,000 (95% UI) | | AAPC | P value | SDI 1990 | SDI 2021 |
| --- | --- | --- | --- | --- | --- | --- |
|  | 1990 | 2021 |  |  |  |  |
| Antigua and Barbuda | 12093.03 (10973.69 to 13138.14) | 6860.87 (6101 to 7873.03) | -1.8939 | <0.001 | 0.61210459 | 0.74988689 |
| Bahamas | 12925.28 (11681.94 to 14138.64) | 7352.93 (5973.4 to 8954.55) | -1.7665 | <0.001 | 0.69350927 | 0.80502067 |
| Barbados | 10788.12 (9861.23 to 11619.51) | 5826.92 (4640.01 to 7157.96) | -1.9315 | <0.001 | 0.65358252 | 0.74674876 |
| Belize | 11619.24 (10623.25 to 12538.67) | 6545.69 (5664.23 to 7453.56) | -1.8403 | <0.001 | 0.42372699 | 0.61022900 |
| Benin | 7272.74 (5512.45 to 9385.72) | 7522.97 (5594.12 to 9869.6) | 0.3043 | 0.103 | 0.21890715 | 0.37348657 |
| Bolivia (Plurinational State of) | 13397.68 (9775.1 to 18504.84) | 7815.2 (5325.33 to 11537.11) | -1.6603 | <0.001 | 0.42391796 | 0.59901080 |
| Botswana | 9837.49 (6992.34 to 13100.64) | 7621.26 (5598 to 10062.43) | -0.9441 | <0.001 | 0.41807775 | 0.64272163 |
| Brunei Darussalam | 14111.31 (11508.67 to 17084.19) | 7158.6 (5832.37 to 8701.35) | -2.1622 | <0.001 | 0.66608192 | 0.81023437 |
| Burundi | 10658.69 (7399.88 to 14570.19) | 8534.78 (6149.73 to 11480.46) | -0.7531 | <0.001 | 0.20586736 | 0.28937436 |
| Cameroon | 7859.11 (5506.94 to 10944.78) | 10078.12 (7000.77 to 14068.12) | 0.9282 | <0.001 | 0.30305533 | 0.47969122 |
| Comoros | 7806.36 (5479.35 to 10710.3) | 7165.14 (5014.98 to 9806.33) | -0.3132 | <0.001 | 0.27004812 | 0.47597869 |
| Cook Islands | 15027.09 (12053.23 to 18354.09) | 9708.85 (7534.95 to 12155.54) | -1.2662 | <0.001 | 0.56451485 | 0.77910995 |
| Djibouti | 7047.63 (4604.07 to 9996.45) | 9130.59 (6280.56 to 12757.58) | 0.8286 | <0.001 | 0.33778179 | 0.48795837 |
| Dominica | 14052.14 (12044.65 to 16199.52) | 8711.64 (6886.73 to 10923.53) | -1.5419 | <0.001 | 0.56360259 | 0.74696718 |
| Ecuador | 8630.97 (7967.87 to 9244.53) | 6620.73 (5325.31 to 8147.14) | -0.5529 | 0.302 | 0.51843061 | 0.66101705 |
| El Salvador | 10931.71 (9521.11 to 12249.43) | 8644.96 (6872.65 to 10671.39) | -0.7302 | <0.001 | 0.37305797 | 0.56377519 |
| Eritrea | 8472.51 (5692.33 to 11635.79) | 8841.63 (6259.11 to 12039.59) | 0.1151 | 0.170 | 0.21602824 | 0.40386394 |
| Ghana | 14009.82 (10706.63 to 17901.55) | 9440.39 (6874.02 to 12338.21) | -1.1558 | <0.001 | 0.37311200 | 0.56493039 |
| Grenada | 14292.41 (12694.05 to 15876.37) | 9422.12 (8063.18 to 10679.82) | -1.4550 | 0.012 | 0.43673442 | 0.66899303 |
| Guatemala | 12893.1 (11953.8 to 13780.71) | 7990.89 (6795.16 to 9195.19) | -1.1952 | 0.001 | 0.31179246 | 0.53997242 |
| Jamaica | 6646.88 (5990 to 7237.53) | 5151.55 (4007.58 to 6490.3) | -0.3180 | 0.354 | 0.53478123 | 0.68326306 |
| Jordan | 19103.96 (15890.89 to 22593.54) | 9045.14 (7235.94 to 11139.57) | -2.6836 | <0.001 | 0.53914747 | 0.72530723 |
| Lebanon | 22077.53 (17737.45 to 27515.21) | 8189.7 (6604.27 to 9956.38) | -3.1362 | <0.001 | 0.53671897 | 0.74474635 |
| Lesotho | 5005.64 (3450.68 to 7078.21) | 8964.48 (5700.07 to 14046.15) | 2.1580 | <0.001 | 0.33915513 | 0.51039307 |
| Malawi | 7031.46 (5287.68 to 9053.57) | 8435.76 (6334.34 to 10865.73) | 0.5761 | <0.001 | 0.20401024 | 0.38455363 |
| Mali | 7481.62 (5188.58 to 10329.32) | 7066.95 (4973.84 to 9663.42) | -0.1675 | 0.043 | 0.12652643 | 0.26857994 |
| Mauritius | 21701.06 (19888.28 to 23294.97) | 8573.35 (7674.39 to 9365.65) | -3.0567 | <0.001 | 0.54458653 | 0.71826045 |
| Mexico | 9905.41 (9473.33 to 10196.1) | 9779.25 (8624.87 to 10950.65) | 0.0549 | 0.614 | 0.50499608 | 0.66457530 |
| Mozambique | 3565.08 (2620.85 to 4786.82) | 4923.05 (3358.76 to 6820.83) | 1.0595 | <0.001 | 0.17306472 | 0.32646261 |
| Namibia | 10364.72 (7664.62 to 13306) | 10056.81 (7305.71 to 13476.39) | 0.0340 | 0.726 | 0.45004023 | 0.61756487 |
| Nicaragua | 8161.32 (6915.95 to 9338.6) | 7357.43 (5909.14 to 8993.54) | -0.2767 | 0.328 | 0.34603523 | 0.52395847 |
| Paraguay | 10335.9 (8738.14 to 12064.7) | 8523.95 (6512.02 to 10813.72) | -0.3718 | 0.062 | 0.46952779 | 0.63571810 |
| Puerto Rico | 14807.44 (13800.74 to 15654.05) | 5163.94 (4248.55 to 6109.89) | -3.3511 | <0.001 | 0.65875815 | 0.82552585 |
| Saint Kitts and Nevis | 20767.97 (19174.43 to 22380.19) | 8665.08 (7172.05 to 10520.13) | -3.1984 | <0.001 | 0.58068588 | 0.75498705 |
| Saint Lucia | 12795.68 (11758.03 to 13814.75) | 4451.55 (3634.8 to 5340.69) | -3.6247 | <0.001 | 0.49629657 | 0.67250974 |
| Saint Vincent and the Grenadines | 15733.44 (14357.51 to 17107.47) | 8674.29 (7574.25 to 9781.13) | -2.4008 | <0.001 | 0.47593019 | 0.63719596 |
| Seychelles | 12591.48 (10659.39 to 14625.71) | 7827.2 (6516.91 to 9285.38) | -1.4522 | <0.001 | 0.57552650 | 0.73015077 |
| Somalia | 6897.81 (4580.24 to 10176.36) | 7119.04 (4578.95 to 10644.52) | 0.1691 | 0.020 | 0.04884856 | 0.07768811 |
| South Africa | 6527.55 (5162.1 to 7558.58) | 7198.84 (6417.68 to 7966.81) | 0.4898 | 0.007 | 0.54157144 | 0.67962660 |
| South Sudan | 7512.91 (5053.39 to 10637.36) | 8316.59 (5593.15 to 11747.84) | 0.3415 | <0.001 | 0.20665650 | 0.27837113 |
| Sri Lanka | 12848.53 (10954.25 to 14913.13) | 8858.45 (5942.72 to 12003.87) | -0.9000 | <0.001 | 0.52262255 | 0.70153494 |
| Suriname | 17036.13 (14769.35 to 19206.84) | 9045.98 (6678.95 to 11778.4) | -1.9356 | 0.005 | 0.50205430 | 0.63366574 |
| Uganda | 7120.35 (5050.23 to 9649.35) | 6994.51 (4977.01 to 9587.2) | -0.1787 | 0.004 | 0.18700110 | 0.42326118 |
| United States Virgin Islands | 19273.36 (16116.57 to 22551.46) | 9048.85 (7090.06 to 11394) | -2.1293 | <0.001 | 0.65516086 | 0.82183085 |
| Viet Nam | 6169.96 (4456.41 to 8313.55) | 7023.26 (5253.24 to 9070.44) | 0.4615 | <0.001 | 0.40763005 | 0.62793372 |
| Zambia | 6268.9 (4535.51 to 8361.22) | 9188.47 (6545.93 to 12389.67) | 1.3606 | <0.001 | 0.30400855 | 0.50594895 |

Table S3. In the ischaemic heart disease-dominant regions, ASDR of ischemic heart disease among people aged 55 years and older in 1990 and 2021, and change from 1990 to 2021. Abbreviations: ASDR, age-standardized disability-adjusted life years rates; SDI, sociodemographic index; UI, uncertainty interval; AAPC, average annual percent change.

| Location | ASDR, per 100,000 (95% UI) | | | AAPC | P value | SDI 1990 | SDI 2021 |
| --- | --- | --- | --- | --- | --- | --- | --- |
|  | 1990 | | 2021 |  |  |  |  |
| Albania | 16206.71 (14047.1 to 18346.07) | 13252.33 (10861.7 to 15881.43) | | -0.6416 | 0.005 | 0.5577733 | 0.7068498 |
| Algeria | 25675.43 (20952.68 to 30844.73) | 16887.51 (13035.04 to 20894.87) | | -1.3581 | <0.001 | 0.4604869 | 0.6595009 |
| Armenia | 27806.56 (25878.2 to 29321.29) | 18748.43 (16547.93 to 20995.99) | | -1.3091 | <0.001 | 0.5444145 | 0.7018332 |
| Azerbaijan | 33567.55 (30705.32 to 36146.87) | 28360.49 (24590.56 to 32146.57) | | -0.5342 | 0.010 | 0.5959860 | 0.6948513 |
| Bangladesh | 12004.04 (9388.84 to 15036.54) | 10299.4 (7765.74 to 13264.85) | | -0.1778 | 0.555 | 0.2285489 | 0.4924209 |
| Belarus | 29018.84 (27027.04 to 30456.62) | 31040.27 (25908.2 to 36399.12) | | 0.1611 | 0.479 | 0.6224466 | 0.7844847 |
| Bhutan | 10716.64 (7307.83 to 14530.49) | 10549.43 (7767.23 to 13637.73) | | -0.0470 | 0.135 | 0.2150399 | 0.4730624 |
| Bulgaria | 33026.83 (31105.47 to 34663.53) | 17860.73 (15381.81 to 20456.32) | | -1.9122 | <0.001 | 0.6334465 | 0.7681509 |
| Chad | 8851.27 (6485.12 to 11854.72) | 10499.43 (7469.48 to 14095.09) | | 0.5508 | <0.001 | 0.1146388 | 0.2404360 |
| Croatia | 24887.19 (23192.59 to 26388.56) | 10901.22 (9475.13 to 12174.37) | | -2.7356 | <0.001 | 0.6689064 | 0.7983410 |
| Czechia | 28498.13 (26997.75 to 29742.28) | 11047.88 (9559.55 to 12304.9) | | -3.1355 | <0.001 | 0.6818480 | 0.8284504 |
| Democratic People's Republic of Korea | 9954.55 (7038.46 to 13241.2) | 11044.67 (8170.52 to 14263.46) | | 0.3052 | <0.001 | 0.4977801 | 0.5698546 |
| Georgia | 32372.66 (30353.58 to 33926.76) | 11653.74 (10310.59 to 12965.38) | | -3.4677 | <0.001 | 0.6561360 | 0.7324736 |
| Guam | 18698.02 (16370.98 to 21045.35) | 12470.15 (10762.8 to 14151.52) | | -1.1065 | 0.003 | 0.6762203 | 0.8039822 |
| Hungary | 23992.82 (22437.67 to 25365.18) | 14178.36 (12332.69 to 15769.49) | | -1.5514 | <0.001 | 0.6494200 | 0.7907548 |
| India | 13680.56 (11982.51 to 15117.42) | 14755.18 (13291.99 to 16309.24) | | 0.1797 | 0.125 | 0.3325936 | 0.5754016 |
| Indonesia | 9860.39 (8110.84 to 11612.24) | 13234.56 (10796.69 to 15439.53) | | 0.9460 | <0.001 | 0.4571350 | 0.6568683 |
| Iran (Islamic Republic of) | 24354.72 (22300.52 to 26067.21) | 13135.47 (11827.53 to 14250.86) | | -1.9098 | <0.001 | 0.4537999 | 0.6972074 |
| Kazakhstan | 26974.68 (24693.83 to 28983.01) | 20955.07 (18572.91 to 23248.73) | | -1.1150 | 0.003 | 0.5894358 | 0.7251445 |
| Kyrgyzstan | 23899.46 (21542.75 to 26055.76) | 24670.89 (20776.46 to 28468.94) | | 0.1915 | 0.362 | 0.5194077 | 0.6039793 |
| Latvia | 30590.23 (28536.85 to 32218.14) | 14542.95 (12662.3 to 16247.46) | | -2.4608 | <0.001 | 0.6801936 | 0.8306635 |
| Lithuania | 31454.33 (29396.13 to 32877.73) | 16862.48 (14734.76 to 18702.31) | | -2.2404 | <0.001 | 0.6685039 | 0.8564840 |
| Malaysia | 18591.56 (15884.17 to 21279.44) | 14463.33 (12382.51 to 16604.8) | | -0.9776 | <0.001 | 0.5457994 | 0.7425238 |
| Mauritania | 13638.17 (10059.3 to 17856.2) | 10791.32 (7709.61 to 14481.7) | | -0.8544 | <0.001 | 0.3357809 | 0.4989451 |
| Mongolia | 29962.34 (25501.62 to 34513.97) | 19098.93 (15930.43 to 22171.74) | | -1.6307 | <0.001 | 0.4665501 | 0.6176216 |
| Montenegro | 15340.73 (13044.24 to 17626.76) | 18334.58 (15465.23 to 21353.25) | | 0.8611 | <0.001 | 0.6742257 | 0.7958006 |
| Nepal | 12942.52 (9668.57 to 16832.81) | 13018.1 (9939.7 to 16802.41) | | -0.0140 | 0.761 | 0.1995607 | 0.4331746 |
| Nigeria | 10256.54 (8023.36 to 12867.09) | 10178.39 (8161.38 to 12389.93) | | 0.2046 | 0.381 | 0.3058680 | 0.5033908 |
| Poland | 24647.73 (23514.05 to 25396.08) | 10278.52 (9117.11 to 11193.11) | | -3.0827 | <0.001 | 0.6272279 | 0.8120428 |
| Republic of Moldova | 35278.38 (33125.58 to 36916.61) | 21911.21 (19590.24 to 24202.71) | | -1.5703 | <0.001 | 0.6042518 | 0.7322149 |
| Romania | 24121.02 (22595.21 to 25400.95) | 13886.1 (12217.9 to 15515.44) | | -1.8631 | <0.001 | 0.6192989 | 0.7684539 |
| Russian Federation | 27594.92 (26316.63 to 28287.24) | 19706.72 (17863.82 to 21340.09) | | -1.0684 | 0.008 | 0.6716006 | 0.8085360 |
| Serbia | 26373.75 (23054.08 to 29590.59) | 15305.53 (12739.03 to 17995.72) | | -1.7304 | <0.001 | 0.6305110 | 0.7924163 |
| Sierra Leone | 12742.46 (9577.84 to 16535.24) | 12669.39 (9230.56 to 16787.2) | | <0.0018 | 0.990 | 0.2115693 | 0.3586659 |
| Slovakia | 30674.96 (28089.32 to 33045.61) | 16151.72 (13671.31 to 18479.43) | | -2.0247 | <0.001 | 0.6538535 | 0.8106105 |
| Sudan | 32914.86 (25639.93 to 41151.01) | 24280.63 (18358.43 to 31971.63) | | -0.9939 | <0.001 | 0.2921786 | 0.5419497 |
| Syrian Arab Republic | 33581.39 (27296.92 to 40497.38) | 31357.02 (24466.29 to 39627.71) | | -0.3234 | 0.004 | 0.4304926 | 0.6230041 |
| Tajikistan | 28530.51 (24629.11 to 31947.73) | 22538.68 (18752.33 to 26220.19) | | -1.1458 | <0.001 | 0.4661554 | 0.5415112 |
| Timor-Leste | 11782.99 (8828.67 to 15392.54) | 14547.41 (10721.78 to 18945.6) | | 0.7272 | <0.001 | 0.2624681 | 0.4446676 |
| Togo | 11330.44 (8691.21 to 14401.42) | 11507.91 (8167.46 to 15691.44) | | 0.0738 | 0.373 | 0.2696923 | 0.4085337 |
| Tunisia | 20015.01 (16176.71 to 23966.82) | 14747.6 (10589.52 to 19965.49) | | -1.0972 | <0.001 | 0.4711385 | 0.6824322 |
| Turkey | 19421.72 (16058.06 to 22920.27) | 11954.43 (9408.48 to 14611.46) | | -1.4069 | <0.001 | 0.4616070 | 0.7126927 |
| Turkmenistan | 37673.05 (35307.86 to 39465.62) | 32422.01 (26039.26 to 39854.6) | | -0.8699 | 0.235 | 0.5631269 | 0.6821608 |
| Ukraine | 29767.81 (27782.68 to 31246.62) | 33373.97 (25405.4 to 41834.62) | | 0.1131 | 0.540 | 0.6474615 | 0.7607739 |
| Uzbekistan | 29092.18 (26795.08 to 30816.58) | 31458.33 (27081.21 to 35998.42) | | 0.2138 | 0.228 | 0.5002417 | 0.6626217 |
| Yemen | 31786.29 (23643.86 to 41731.88) | 25260.45 (18410.66 to 33233.8) | | -0.7623 | <0.001 | 0.2156646 | 0.4503764 |

Table S4. In the dual-burden regions, ASDR of ischemic heart disease among people aged 55 years and older in 1990 and 2021, and change from 1990 to 2021. Abbreviations: ASDR, age-standardized disability-adjusted life years rates; SDI, sociodemographic index; UI, uncertainty interval; AAPC, average annual percent change.

| Location | ASDR, per 100,000 (95% UI) | | | AAPC | P value | SDI 1990 | SDI 2021 |
| --- | --- | --- | --- | --- | --- | --- | --- |
|  | 1990 | | 2021 |  |  |  |  |
| Afghanistan | 36329.48 (26705.91 to 46731.44) | 26893.14 (20044.75 to 34171.7) | | -0.9750 | <0.001 | 0.1738322 | 0.3372000 |
| American Samoa | 15335.2 (12651.68 to 18232.17) | 16234.35 (13298.3 to 19661.85) | | 0.2982 | <0.001 | 0.6136339 | 0.7237275 |
| Angola | 13170.04 (9631.24 to 17585.24) | 12438.42 (9039.81 to 16490.48) | | -0.1796 | 0.003 | 0.2707369 | 0.4537219 |
| Bahrain | 38010.41 (34112.7 to 41857.62) | 13966.71 (11553.18 to 16596.17) | | -3.3311 | <0.001 | 0.5845789 | 0.7530432 |
| Bosnia and Herzegovina | 19175.3 (17015.24 to 21198.06) | 12555.27 (10094.95 to 14969.14) | | -1.3054 | <0.001 | 0.5411325 | 0.7230779 |
| Cabo Verde | 6705.94 (5144.98 to 8359.83) | 11248.93 (8722.32 to 14112.22) | | 1.8900 | <0.001 | 0.2767234 | 0.5335345 |
| Cambodia | 11405.06 (8699.9 to 14722.32) | 10404.69 (7662.57 to 13508.01) | | -0.2897 | <0.001 | 0.2890751 | 0.4736215 |
| Central African Republic | 17255.43 (12137.89 to 24566.87) | 15232.35 (10373.58 to 22024.49) | | -0.4017 | <0.001 | 0.2168252 | 0.3091677 |
| Congo | 18707.29 (13936.44 to 24367.61) | 15584.79 (11758.22 to 20052.02) | | -0.5607 | <0.001 | 0.4206547 | 0.5830752 |
| Coted'Ivoire | 11733.34 (8539.61 to 15335.52) | 11667.53 (8594.62 to 15530.37) | | 0.0771 | 0.409 | 0.2793204 | 0.4259419 |
| Democratic Republic of the Congo | 13310.44 (9395.71 to 18166.22) | 11273.03 (7804.7 to 15608.47) | | -0.5773 | <0.001 | 0.2898432 | 0.3831798 |
| Dominican Republic | 13179.49 (11213.07 to 15425.46) | 13628.4 (10566.92 to 17239.66) | | 0.1843 | 0.367 | 0.4426541 | 0.6193882 |
| Egypt | 34086.89 (28735.19 to 39581.61) | 32229.72 (26455.3 to 38550.96) | | -0.2076 | 0.160 | 0.4171827 | 0.6067871 |
| Equatorial Guinea | 16505.13 (11618.99 to 22297.25) | 13686.44 (9213.65 to 18990.58) | | -0.5827 | <0.001 | 0.2687836 | 0.6578575 |
| Eswatini | 8941.51 (6400.07 to 11986.83) | 10416.69 (6893.29 to 15313.8) | | 0.6287 | <0.001 | 0.3994210 | 0.5854597 |
| Fiji | 27599 (22653.97 to 33011.04) | 24758.17 (19028.79 to 31212.44) | | -0.2921 | 0.033 | 0.5346489 | 0.6750516 |
| Gabon | 13470.99 (9841.49 to 17531.58) | 12283.43 (8769.99 to 16116.78) | | -0.2118 | <0.001 | 0.4554212 | 0.6346914 |
| Gambia | 11200.68 (8183.69 to 14952.21) | 13648.89 (9835.75 to 17927.25) | | 0.7293 | <0.001 | 0.2387148 | 0.4097142 |
| Guinea | 9197.07 (6656.08 to 12184.72) | 10899.35 (7758.21 to 14784.98) | | 0.6741 | <0.001 | 0.1782954 | 0.3364013 |
| Guinea-Bissau | 15517.61 (11275.28 to 21117.54) | 15523.81 (11435.81 to 20229.71) | | 0.0651 | 0.356 | 0.2076148 | 0.3531096 |
| Guyana | 22949.58 (20297.46 to 25737.05) | 14352.7 (11215.81 to 18155.13) | | -1.5459 | 0.025 | 0.4604301 | 0.6508123 |
| Haiti | 26145.07 (20778.02 to 32009.64) | 20674.51 (14956.45 to 27411.47) | | -0.7093 | <0.001 | 0.3103346 | 0.4482783 |
| Honduras | 10377.57 (8271.69 to 12639.38) | 15677.84 (12236.26 to 19798.11) | | 1.3277 | 0.031 | 0.3320429 | 0.5130372 |
| Iraq | 25127.35 (20646.44 to 30160.71) | 24247.09 (18542.29 to 29490.91) | | -0.0673 | 0.553 | 0.4120442 | 0.6626262 |
| Kiribati | 18296.26 (13985.08 to 23061.74) | 18726.61 (14452.82 to 24101.21) | | 0.0498 | 0.439 | 0.4103898 | 0.5271866 |
| Kuwait | 19576.96 (17579.29 to 21300.34) | 10291.18 (8324.35 to 12533.16) | | -2.4303 | 0.005 | 0.6645179 | 0.8466511 |
| Lao People's Democratic Republic | 22874.77 (17128.25 to 29366.98) | 16265.1 (12353.19 to 20783.03) | | -1.0987 | <0.001 | 0.2642832 | 0.4891361 |
| Liberia | 10600.27 (8095.69 to 13538.49) | 11120 (8147.54 to 15131.02) | | 0.2369 | 0.012 | 0.2352968 | 0.3524425 |
| Libya | 15303.28 (11734.86 to 19100.38) | 16688.58 (12643.07 to 21730.23) | | 0.3516 | 0.041 | 0.5279982 | 0.7257714 |
| Marshall Islands | 25059.13 (19836.73 to 30779.38) | 25107.72 (19093.64 to 32237.25) | | -0.0552 | 0.516 | 0.4308393 | 0.5740911 |
| Micronesia (Federated States of) | 24898.75 (19141.07 to 31484.57) | 24123.39 (18386.64 to 31461.19) | | -0.0997 | <0.001 | 0.4625116 | 0.5875350 |
| Morocco | 28263.9 (23519.41 to 33527.74) | 25193.1 (19360.63 to 30217.71) | | -0.3543 | <0.001 | 0.3580729 | 0.5626983 |
| Myanmar | 18506.23 (13863.24 to 24063.17) | 12714.49 (9605.13 to 16577.34) | | -1.2644 | <0.001 | 0.3192197 | 0.5339008 |
| Nauru | 38325.14 (29731.91 to 47725.05) | 39483.92 (30514.36 to 49702.97) | | 0.0806 | 0.073 | 0.5391460 | 0.6251778 |
| Niue | 22385.04 (17763.79 to 27390.76) | 22362.79 (17911.28 to 26869.25) | | -0.0379 | 0.191 | 0.5875330 | 0.7262220 |
| North Macedonia | 20316.2 (17683.98 to 23058.1) | 16454.66 (13749.61 to 19462.31) | | -0.5748 | <0.001 | 0.6090261 | 0.7506297 |
| Northern Mariana Islands | 12452.83 (9796.99 to 15827.72) | 14065.18 (11514.05 to 16481.47) | | 0.4778 | <0.001 | 0.7085938 | 0.7715352 |
| Oman | 31789 (24256.12 to 40618.59) | 17132.14 (13596.5 to 21086.15) | | -1.9287 | <0.001 | 0.4292709 | 0.7733916 |
| Pakistan | 13772.98 (10802.38 to 16457.06) | 17509 (14292.27 to 21668.57) | | 0.9126 | <0.001 | 0.3104676 | 0.5040287 |
| Palau | 23012.74 (18321.59 to 28464.84) | 20092.36 (15823.2 to 24875.39) | | -0.4472 | <0.001 | 0.6629095 | 0.7540469 |
| Palestine | 26920.42 (21120.89 to 33492.33) | 17219.83 (14294.32 to 20259.79) | | -1.5573 | <0.001 | 0.4017922 | 0.6310117 |
| Papua New Guinea | 13334.34 (9507.92 to 18311.25) | 13919.1 (9987.08 to 18628.21) | | 0.1096 | 0.003 | 0.3106686 | 0.4177974 |
| Philippines | 14502.51 (12864.08 to 15956.37) | 13720.78 (11604.06 to 15912.28) | | -0.0662 | 0.466 | 0.5100118 | 0.6512193 |
| Qatar | 36599.44 (30226.68 to 43521.98) | 11003.65 (8262.37 to 14248.67) | | -3.6960 | <0.001 | 0.6512084 | 0.8468606 |
| Samoa | 19381.56 (15228.72 to 23858.85) | 20778.85 (16853.68 to 25555.65) | | 0.2415 | <0.001 | 0.4874914 | 0.5933928 |
| Saudi Arabia | 22110.32 (16429.18 to 28361.24) | 17684.03 (14440.23 to 21370.21) | | -0.8027 | <0.001 | 0.5389545 | 0.8151435 |
| Senegal | 12697.07 (9508.96 to 16240.84) | 11737.45 (8690.04 to 15624.42) | | -0.2953 | 0.005 | 0.2380476 | 0.4080542 |
| Solomon Islands | 28231.17 (20368.67 to 36326.33) | 25649.17 (20201.02 to 32877.22) | | -0.3541 | <0.001 | 0.3012172 | 0.4293603 |
| Tokelau | 21415.72 (16647.99 to 26858.99) | 18707.94 (14325.39 to 23446.85) | | -0.4559 | <0.001 | 0.5219424 | 0.6864256 |
| Tonga | 13390.75 (10609 to 16492.62) | 13015.57 (10051.49 to 16245.36) | | -0.0586 | 0.859 | 0.4918068 | 0.6263499 |
| Trinidad and Tobago | 21759.39 (20380.88 to 22992.31) | 10089.8 (7858.65 to 12745.12) | | -2.4788 | <0.001 | 0.6239702 | 0.7687633 |
| Tuvalu | 26279.66 (21109.07 to 32127.91) | 24341.51 (19812.38 to 29601.96) | | -0.2442 | <0.001 | 0.4062476 | 0.5766205 |
| United Arab Emirates | 27662.93 (21454.2 to 34738.99) | 15673.05 (12251.39 to 19257.09) | | -1.5552 | 0.006 | 0.6444123 | 0.8493177 |
| Vanuatu | 29278.66 (23348.79 to 36793.38) | 28159.99 (22812.66 to 34173.46) | | -0.1805 | <0.001 | 0.3531003 | 0.4731007 |
| Venezuela (Bolivarian Republic of) | 16352.41 (15191.05 to 17280.47) | 13389.99 (10362.41 to 16918.58) | | -0.7387 | <0.001 | 0.5168912 | 0.5965131 |
| Zimbabwe | 7897.9 (5897.93 to 10136.62) | 11480.34 (8667.06 to 15060.81) | | 1.3170 | <0.001 | 0.3985593 | 0.4738195 |

Table S5. In low-burden regions, ASDR of type 2 diabetes mellitus among people aged 55 years and older in 1990 and 2021, and change from 1990 to 2021. Abbreviations: ASDR, age-standardized disability-adjusted life years rates; SDI, sociodemographic index; UI, uncertainty interval; AAPC, average annual percent change.

| Location | ASDR, per 100,000 (95% UI) | | | AAPC | P value | SDI 1990 | SDI 2021 |
| --- | --- | --- | --- | --- | --- | --- | --- |
|  | 1990 | | 2021 |  |  |  |  |
| Andorra | 1670.61 (1229.37 to 2232.82) | 1855.28 (1388.46 to 2483.93) | | 0.2155 | 0.201 | 0.76146388 | 0.8694441 |
| Argentina | 3259.61 (2845.6 to 3733.64) | 3390.36 (2755.94 to 4167.59) | | 0.1175 | 0.352 | 0.58739728 | 0.7231230 |
| Australia | 1704.61 (1448.3 to 2001.71) | 1911.75 (1474.64 to 2428.68) | | 0.2698 | 0.433 | 0.72598252 | 0.8442528 |
| Austria | 1730.13 (1502.27 to 1976.46) | 1632.65 (1305.17 to 2017.79) | | -0.2362 | 0.671 | 0.74985369 | 0.8538370 |
| Belgium | 1695.63 (1390.27 to 2029.36) | 1779.97 (1307.66 to 2366.59) | | 0.0557 | 0.809 | 0.73739066 | 0.8536540 |
| Bermuda | 4755.71 (4145.95 to 5463.31) | 3754.56 (2957.29 to 4738.72) | | -0.8231 | <0.001 | 0.69645120 | 0.8213654 |
| Brazil | 4635.44 (4122.63 to 5216.38) | 4545.76 (3868.23 to 5327.22) | | -0.0364 | 0.790 | 0.50007051 | 0.6530439 |
| Burkina Faso | 4041.02 (3080.63 to 5232.82) | 4690.99 (3558.49 to 6033.13) | | 0.5588 | <0.001 | 0.12969562 | 0.2851184 |
| Canada | 1819.89 (1544.36 to 2146.06) | 2579.29 (1914.87 to 3389.66) | | 1.0018 | <0.001 | 0.78197786 | 0.8731707 |
| Chile | 2599.01 (2219.83 to 3041.5) | 3234.71 (2457.8 to 4186.23) | | 0.4758 | 0.106 | 0.58649510 | 0.7715147 |
| China | 1796.74 (1497.74 to 2161.27) | 2034.75 (1625.82 to 2528.03) | | 0.3944 | <0.001 | 0.45866894 | 0.7216298 |
| Colombia | 3549.12 (2977.5 to 4226.84) | 3320.72 (2601.78 to 4186.35) | | -0.3429 | 0.095 | 0.48072005 | 0.6554429 |
| Costa Rica | 3004.18 (2466.16 to 3665.02) | 4310.47 (3414.67 to 5413.18) | | 1.0555 | <0.001 | 0.53412518 | 0.7003405 |
| Cuba | 3497 (2989.19 to 4087.62) | 3138.99 (2393.01 to 4043.93) | | -0.3732 | 0.016 | 0.55801907 | 0.6687299 |
| Cyprus | 7754.33 (6086.91 to 9652.6) | 3962.24 (3075.06 to 5010.65) | | -2.2461 | <0.001 | 0.64823087 | 0.8356305 |
| Denmark | 1287.45 (1124.61 to 1467.55) | 1707.26 (1395.56 to 2070.11) | | 0.7281 | 0.060 | 0.80115466 | 0.8964242 |
| Estonia | 1071.33 (838.85 to 1335.74) | 2370.08 (1895.7 to 2985.46) | | 2.6398 | <0.001 | 0.67496763 | 0.8449178 |
| Ethiopia | 7672.23 (6351.06 to 9276.79) | 4799.56 (3995.76 to 5688.96) | | -1.5373 | <0.001 | 0.14803388 | 0.3588233 |
| Finland | 1303.09 (1038.1 to 1622.91) | 1728.07 (1260.49 to 2286.6) | | 0.8829 | <0.001 | 0.75622151 | 0.8598314 |
| France | 1202.55 (1019.53 to 1402.78) | 1347.99 (1066.7 to 1686.19) | | 0.3023 | 0.133 | 0.73074747 | 0.8383649 |
| Germany | 1807.72 (1563.18 to 2070.14) | 1849.16 (1468.86 to 2306.93) | | 0.1057 | 0.702 | 0.81707767 | 0.9029571 |
| Greece | 1561.87 (1231.43 to 1945.04) | 1937.33 (1455.07 to 2525.58) | | 0.5928 | 0.005 | 0.67418647 | 0.7918544 |
| Greenland | 1747.05 (1339.68 to 2253.81) | 2011.46 (1523.52 to 2581.25) | | 0.4922 | <0.001 | 0.73225825 | 0.8262103 |
| Iceland | 974.56 (804.76 to 1189.91) | 1425.96 (1060.6 to 1885.99) | | 1.2578 | <0.001 | 0.76421252 | 0.8763617 |
| Ireland | 1595.78 (1350 to 1882.56) | 1350.52 (1006.8 to 1775.54) | | -0.6976 | <0.001 | 0.71989182 | 0.8737538 |
| Israel | 2974.48 (2554.83 to 3451.96) | 3057.27 (2443.5 to 3749.67) | | -0.0572 | 0.820 | 0.70917835 | 0.8090117 |
| Italy | 2653.74 (2346.35 to 3000.29) | 2107.02 (1694.73 to 2616.66) | | -0.8405 | <0.001 | 0.70625522 | 0.8057735 |
| Japan | 1708.73 (1379.42 to 2130.41) | 1937.27 (1394.19 to 2656.22) | | 0.3564 | 0.054 | 0.79025352 | 0.8712418 |
| Kenya | 3193.84 (2642.25 to 3877.77) | 4422.29 (3694.93 to 5292.18) | | 1.0458 | <0.001 | 0.33385029 | 0.5237681 |
| Luxembourg | 1601.49 (1356.01 to 1898.24) | 1617.6 (1238.18 to 2093.3) | | -0.0233 | 0.914 | 0.78105161 | 0.8844290 |
| Madagascar | 4025.41 (2982.11 to 5352.09) | 4429.8 (3232.05 to 5923.65) | | 0.3363 | 0.007 | 0.27988946 | 0.4002469 |
| Maldives | 4653.84 (3637.36 to 5876.86) | 3871.48 (2986.94 to 4952.15) | | -0.6906 | <0.001 | 0.33160154 | 0.6508866 |
| Malta | 2995.24 (2598.38 to 3435.99) | 2879.32 (2273.24 to 3666.15) | | -0.3546 | 0.258 | 0.65650458 | 0.8015850 |
| Monaco | 750.42 (566.25 to 978.64) | 1263.94 (925.17 to 1694.94) | | 1.7182 | <0.001 | 0.84549515 | 0.9082628 |
| Netherlands | 2336.25 (1990.73 to 2698.88) | 1677.37 (1285.09 to 2128.68) | | -0.9247 | <0.001 | 0.79461212 | 0.8884643 |
| New Zealand | 1924.3 (1624.33 to 2297.69) | 2005.96 (1578.18 to 2515.19) | | 0.2522 | 0.073 | 0.75232165 | 0.8494425 |
| Niger | 2988.73 (2197.86 to 3986.92) | 4325.42 (3223.94 to 5757.78) | | 1.2926 | <0.001 | 0.08086848 | 0.1680728 |
| Norway | 1353.99 (1112.91 to 1663.32) | 1426.09 (1107.7 to 1846.96) | | 0.2341 | 0.111 | 0.79588728 | 0.9161328 |
| Panama | 3373.71 (2848.02 to 4022.14) | 5253.35 (4192.03 to 6545.74) | | 1.6014 | <0.001 | 0.54604812 | 0.7088648 |
| Peru | 2160.2 (1738.31 to 2642.72) | 3153.33 (2437.07 to 4028.59) | | 1.3426 | <0.001 | 0.51041985 | 0.6620540 |
| Portugal | 3030.11 (2612.16 to 3486.06) | 2942.01 (2258.97 to 3790.88) | | -0.2902 | 0.327 | 0.59977776 | 0.7441519 |
| Republic of Korea | 3266.3 (2631.51 to 3984.12) | 3663.54 (2686.23 to 4855.72) | | 0.3837 | <0.001 | 0.69232931 | 0.8866753 |
| Rwanda | 6389.08 (4375.33 to 8965.18) | 4917.86 (3221.99 to 7043.13) | | -0.9690 | <0.001 | 0.27509719 | 0.4355887 |
| San Marino | 1203.29 (930.44 to 1536.64) | 1414.19 (1021.27 to 1918.33) | | 0.4407 | <0.001 | 0.81324489 | 0.8880055 |
| Sao Tome and Principe | 2189.56 (1721.14 to 2766.76) | 3710.35 (2882.59 to 4785.42) | | 1.7255 | <0.001 | 0.30954285 | 0.5054137 |
| Singapore | 3529.94 (2901.97 to 4301.08) | 2341.58 (1601.91 to 3297.19) | | -1.1888 | <0.001 | 0.68640444 | 0.8560978 |
| Slovenia | 2305.55 (1868.02 to 2843.01) | 2341.02 (1806.79 to 3012.33) | | -0.5068 | 0.258 | 0.72746393 | 0.8424307 |
| Spain | 2907.72 (2378.23 to 3523.2) | 2552.94 (1864.77 to 3429.36) | | -0.4343 | <0.001 | 0.63667317 | 0.7692837 |
| Sweden | 1422.11 (1182.34 to 1712.53) | 1691.51 (1329.97 to 2187.02) | | 0.5097 | <0.001 | 0.78553579 | 0.8868803 |
| Switzerland | 2140.95 (1781.23 to 2564.84) | 2132.05 (1558.24 to 2856.38) | | 0.0189 | 0.853 | 0.86276684 | 0.9330591 |
| Taiwan (Province of China) | 4832.33 (4243.82 to 5472.08) | 4211.6 (3411.7 to 5129.06) | | -0.6455 | 0.016 | 0.66763385 | 0.8747471 |
| Thailand | 3457.76 (2711.21 to 4382.46) | 4269.8 (3310.65 to 5467.92) | | 0.5064 | <0.001 | 0.50664486 | 0.6825479 |
| United Kingdom | 1459.42 (1251.36 to 1726.49) | 1800.38 (1356.34 to 2372.01) | | 0.5997 | 0.009 | 0.74433413 | 0.8590002 |
| United Republic of Tanzania | 4154.65 (3149.69 to 5344.87) | 4672.89 (3513.29 to 6169.34) | | 0.3816 | <0.001 | 0.25930607 | 0.4465683 |
| United States of America | 2476.58 (2146.12 to 2895.26) | 3780.63 (2985.52 to 4762.6) | | 1.3787 | <0.001 | 0.76364769 | 0.8624484 |
| Uruguay | 2383.12 (2071.08 to 2741.03) | 3300.97 (2672.11 to 4056.91) | | 1.0337 | <0.001 | 0.58192186 | 0.7192834 |

Table S6. In the type 2 diabetes mellitus-dominant regions, ASDR of type 2 diabetes mellitus among people aged 55 years and older in 1990 and 2021, and change from 1990 to 2021. Abbreviations: ASDR, age-standardized disability-adjusted life years rates; SDI, sociodemographic index; UI, uncertainty interval; AAPC, average annual percent change.

| Location | ASDR, per 100,000 (95% UI) | | | AAPC | P value | SDI 1990 | SDI 2021 |
| --- | --- | --- | --- | --- | --- | --- | --- |
|  | 1990 | | 2021 |  |  |  |  |
| Antigua and Barbuda | 8872.16 (7791.84 to 10123.31) | 9642.78 (8118.75 to 11489.67) | | 0.2115 | 0.183 | 0.61210459 | 0.74988689 |
| Bahamas | 6939.7 (5973.83 to 7980.88) | 6839.13 (5488.9 to 8478.55) | | -0.0326 | 0.764 | 0.69350927 | 0.80502067 |
| Barbados | 9707.09 (8562.43 to 11005.85) | 9125.11 (7447.34 to 11096.81) | | -0.2454 | 0.358 | 0.65358252 | 0.74674876 |
| Belize | 6520.04 (5736.5 to 7405.47) | 8594.31 (7277.41 to 10158.24) | | 0.7604 | 0.005 | 0.42372699 | 0.61022900 |
| Benin | 3728.73 (2901.55 to 4719.72) | 5636.07 (4333.66 to 7286.26) | | 1.2816 | <0.001 | 0.21890715 | 0.37348657 |
| Bolivia (Plurinational State of) | 5314.47 (4118.89 to 6847.96) | 6731.68 (5181.5 to 8743.85) | | 0.7444 | <0.001 | 0.42391796 | 0.59901080 |
| Botswana | 7105.2 (5119.15 to 9583.69) | 8235.09 (6373.95 to 10590.17) | | 0.3427 | 0.227 | 0.41807775 | 0.64272163 |
| Brunei Darussalam | 10451.85 (8332.89 to 12836.84) | 8810.1 (6900.53 to 11054.68) | | -0.6024 | 0.005 | 0.66608192 | 0.81023437 |
| Burundi | 6001.48 (4247.11 to 8234.83) | 5449.39 (3803.8 to 7762.77) | | -0.3635 | <0.001 | 0.20586736 | 0.28937436 |
| Cameroon | 4591.58 (3436.95 to 5968) | 6660.35 (4900.54 to 8921.96) | | 1.2071 | <0.001 | 0.30305533 | 0.47969122 |
| Comoros | 5262.96 (3795.9 to 7138.08) | 5811.63 (4251.42 to 7634.96) | | 0.2762 | <0.001 | 0.27004812 | 0.47597869 |
| Cook Islands | 17942.74 (14418.31 to 21914.35) | 16073.11 (12917.08 to 19662.19) | | -0.3638 | <0.001 | 0.56451485 | 0.77910995 |
| Djibouti | 4167.87 (2988.35 to 5748.42) | 5696.51 (4153.24 to 7682.55) | | 0.9859 | <0.001 | 0.33778179 | 0.48795837 |
| Dominica | 9543.76 (7949.18 to 11416.59) | 11089.19 (8877.22 to 13824.43) | | 0.4439 | <0.001 | 0.56360259 | 0.74696718 |
| Ecuador | 3460.49 (2974.55 to 4008.53) | 5514.53 (4385.61 to 6773.5) | | 1.3958 | <0.001 | 0.51843061 | 0.66101705 |
| El Salvador | 3245.39 (2625.89 to 3922.89) | 6515.04 (5093.5 to 8199.5) | | 2.3363 | <0.001 | 0.37305797 | 0.56377519 |
| Eritrea | 5825.68 (4187.77 to 7889.11) | 6707.14 (4919.86 to 8945.25) | | 0.4622 | <0.001 | 0.21602824 | 0.40386394 |
| Ghana | 3436.78 (2624.39 to 4449.67) | 6449.25 (4835.3 to 8413.94) | | 2.1049 | <0.001 | 0.37311200 | 0.56493039 |
| Grenada | 10450.47 (9061.55 to 11843.05) | 12180.78 (10224.25 to 14317.54) | | 0.4883 | 0.412 | 0.43673442 | 0.66899303 |
| Guatemala | 2929.4 (2463 to 3456.17) | 9329.69 (7871.69 to 10954.53) | | 3.8121 | <0.001 | 0.31179246 | 0.53997242 |
| Jamaica | 8933.36 (7999.22 to 9991.21) | 9577.9 (7753.57 to 11799.86) | | 0.1334 | 0.518 | 0.53478123 | 0.68326306 |
| Jordan | 7816.77 (6194.04 to 9751.92) | 7699.72 (5970.08 to 9774.74) | | -0.1443 | 0.379 | 0.53914747 | 0.72530723 |
| Lebanon | 5490.37 (4304.26 to 7038.68) | 6532.37 (4905.28 to 8390.46) | | 0.5809 | <0.001 | 0.53671897 | 0.74474635 |
| Lesotho | 5638.87 (4189.78 to 7454.01) | 12709.16 (9205.86 to 17016.28) | | 2.8811 | <0.001 | 0.33915513 | 0.51039307 |
| Malawi | 4961.97 (3746.78 to 6456.58) | 5539.54 (4239.7 to 7165.83) | | 0.3830 | <0.001 | 0.20401024 | 0.38455363 |
| Mali | 4702.88 (3663.1 to 5985.9) | 7071.53 (5427.45 to 9002.13) | | 1.3495 | <0.001 | 0.12652643 | 0.26857994 |
| Mauritius | 7463.24 (6551.53 to 8502.54) | 14979.32 (13179.11 to 17013.27) | | 2.3467 | <0.001 | 0.54458653 | 0.71826045 |
| Mexico | 9911.71 (9041.53 to 10941.33) | 9822.61 (8602.28 to 11326.77) | | -0.1324 | 0.778 | 0.50499608 | 0.66457530 |
| Mozambique | 4421.33 (3339.08 to 5783.99) | 6082.74 (4493.86 to 8008.06) | | 1.1224 | <0.001 | 0.17306472 | 0.32646261 |
| Namibia | 7409.96 (5699.33 to 9578.19) | 9307.07 (6876.55 to 12122.38) | | 0.8709 | <0.001 | 0.45004023 | 0.61756487 |
| Nicaragua | 4103.66 (3335.73 to 4990.5) | 6075.04 (4804.4 to 7551.74) | | 1.0963 | <0.001 | 0.34603523 | 0.52395847 |
| Paraguay | 3787.59 (3061.06 to 4651.97) | 8385.31 (6567.08 to 10638.74) | | 2.6727 | <0.001 | 0.46952779 | 0.63571810 |
| Puerto Rico | 7490.91 (6504.76 to 8628.6) | 7846.75 (6351.33 to 9706.16) | | 0.0218 | 0.851 | 0.65875815 | 0.82552585 |
| Saint Kitts and Nevis | 9726.5 (8548.07 to 11092.41) | 8841.55 (7193.77 to 10668.19) | | 0.0212 | 0.861 | 0.58068588 | 0.75498705 |
| Saint Lucia | 12521.25 (11032.21 to 14242.59) | 9616.57 (7842.41 to 11760.09) | | -0.9909 | <0.001 | 0.49629657 | 0.67250974 |
| Saint Vincent and the Grenadines | 12193.49 (10728.16 to 13855.54) | 10997.26 (9274.81 to 13104.93) | | -0.4479 | 0.193 | 0.47593019 | 0.63719596 |
| Seychelles | 3025.75 (2381.3 to 3762.39) | 6592.47 (5047.89 to 8482.36) | | 2.5085 | <0.001 | 0.57552650 | 0.73015077 |
| Somalia | 5982.53 (4273.08 to 8101.88) | 6688.33 (4963.7 to 8776.82) | | 0.3997 | <0.001 | 0.04884856 | 0.07768811 |
| South Africa | 5446.66 (4759.49 to 6206.96) | 10442.65 (9330.12 to 11639.38) | | 2.4029 | <0.001 | 0.54157144 | 0.67962660 |
| South Sudan | 5298.33 (3676.52 to 7546.3) | 6748.45 (4777.86 to 9467.48) | | 0.7785 | <0.001 | 0.20665650 | 0.27837113 |
| Sri Lanka | 5508.43 (4405.49 to 6758.31) | 8814.55 (6669.93 to 11297.62) | | 1.5884 | <0.001 | 0.52262255 | 0.70153494 |
| Suriname | 6131.41 (5012.83 to 7356.25) | 8441.95 (6542.41 to 10695.43) | | 1.2141 | <0.001 | 0.50205430 | 0.63366574 |
| Uganda | 4722.71 (3179.59 to 7351.9) | 5542.08 (3887.71 to 7864.65) | | 0.4407 | <0.001 | 0.18700110 | 0.42326118 |
| United States Virgin Islands | 6870.07 (5449.38 to 8483.48) | 7491.77 (5667.66 to 9775.21) | | 0.4188 | <0.001 | 0.65516086 | 0.82183085 |
| Viet Nam | 3876.55 (2940.34 to 5113.94) | 5291.73 (4029.04 to 6754.49) | | 1.0155 | <0.001 | 0.40763005 | 0.62793372 |
| Zambia | 5769.38 (4381.32 to 7550.01) | 6180.54 (4605.12 to 8037.69) | | 0.1360 | 0.037 | 0.30400855 | 0.50594895 |

Table S7. In the ischaemic heart disease-dominant regions, ASDR of type 2 diabetes mellitus among people aged 55 years and older in 1990 and 2021, and change from 1990 to 2021. Abbreviations: ASDR, age-standardized disability-adjusted life years rates; SDI, sociodemographic index; UI, uncertainty interval; AAPC, average annual percent change.

| Location | ASDR, per 100,000 (95% UI) | | | AAPC | P value | SDI 1990 | SDI 2021 |
| --- | --- | --- | --- | --- | --- | --- | --- |
|  | 1990 | | 2021 |  |  |  |  |
| Albania | 1365.55 (1031.59 to 1762.49) | 1727.24 (1246.26 to 2300.88) | | 0.9457 | 0.001 | 0.5577733 | 0.7068498 |
| Algeria | 2395.41 (1846.63 to 3111.8) | 4805.25 (3611.86 to 6324.1) | | 2.2870 | 0.000 | 0.4604869 | 0.6595009 |
| Armenia | 3018.82 (2631.05 to 3470.13) | 3023.7 (2439.69 to 3727.21) | | -0.5113 | 0.168 | 0.5444145 | 0.7018332 |
| Azerbaijan | 1875.5 (1501.4 to 2339.61) | 3513.94 (2652.41 to 4510.91) | | 2.1588 | 0.000 | 0.5959860 | 0.6948513 |
| Bangladesh | 3335.12 (2630.31 to 4158.28) | 4543.24 (3495.16 to 5813.46) | | 1.1694 | 0.000 | 0.2285489 | 0.4924209 |
| Belarus | 939.06 (754.71 to 1154.98) | 1228.79 (937.35 to 1602.13) | | 0.9185 | 0.000 | 0.6224466 | 0.7844847 |
| Bhutan | 3097.16 (2266.74 to 4119.45) | 4451.43 (3309.23 to 5818.56) | | 1.1764 | 0.000 | 0.2150399 | 0.4730624 |
| Bulgaria | 2881.42 (2392.11 to 3412.89) | 3638.33 (2881.26 to 4556.31) | | 0.7688 | 0.000 | 0.6334465 | 0.7681509 |
| Chad | 3069.8 (2296.92 to 4090.2) | 5189.02 (3939.76 to 6829.12) | | 1.7296 | 0.000 | 0.1146388 | 0.2404360 |
| Croatia | 2548.5 (2062.95 to 3125.96) | 3349.85 (2616.14 to 4210.53) | | 0.7475 | 0.018 | 0.6689064 | 0.7983410 |
| Czechia | 2594.82 (2132.08 to 3118.77) | 3780.82 (2994.83 to 4684.51) | | 1.3723 | 0.000 | 0.6818480 | 0.8284504 |
| Democratic People's Republic of Korea | 2124.79 (1566.42 to 2767.9) | 2899.14 (2193.31 to 3783.75) | | 0.9958 | 0.000 | 0.4977801 | 0.5698546 |
| Georgia | 1906.5 (1606.71 to 2265.52) | 3488.5 (2821.42 to 4294.22) | | 1.8999 | 0.051 | 0.6561360 | 0.7324736 |
| Guam | 5681.29 (4535.14 to 6947.54) | 4255.75 (3341.83 to 5375.91) | | -0.9483 | 0.000 | 0.6762203 | 0.8039822 |
| Hungary | 2527.7 (2057.02 to 3050.37) | 3259.23 (2559.78 to 4101.92) | | 1.0081 | 0.016 | 0.6494200 | 0.7907548 |
| India | 3114.59 (2677.2 to 3569.85) | 4533.01 (3918.32 to 5230.74) | | 1.3406 | 0.000 | 0.3325936 | 0.5754016 |
| Indonesia | 2903.92 (2414.9 to 3438.29) | 4632.43 (3854.66 to 5440.96) | | 1.5137 | 0.000 | 0.4571350 | 0.6568683 |
| Iran (Islamic Republic of) | 2331.99 (1924.97 to 2826.62) | 4466.84 (3680.07 to 5454.81) | | 2.1350 | 0.000 | 0.4537999 | 0.6972074 |
| Kazakhstan | 1620.89 (1301.03 to 2014.76) | 2821.76 (2157.13 to 3630.18) | | 1.9289 | 0.000 | 0.5894358 | 0.7251445 |
| Kyrgyzstan | 1135.05 (923.85 to 1388.98) | 2082.15 (1619.92 to 2633.75) | | 2.1383 | 0.000 | 0.5194077 | 0.6039793 |
| Latvia | 1189.18 (964.54 to 1457.02) | 2675.75 (2163.77 to 3319.12) | | 2.3748 | 0.000 | 0.6801936 | 0.8306635 |
| Lithuania | 909.96 (716.9 to 1142.82) | 2040.46 (1637.19 to 2548.05) | | 2.3901 | 0.000 | 0.6685039 | 0.8564840 |
| Malaysia | 4404.7 (3513.77 to 5395.2) | 4647.14 (3568.59 to 5879.53) | | 0.2321 | 0.065 | 0.5457994 | 0.7425238 |
| Mauritania | 3688.44 (2767.35 to 4804.6) | 4736.15 (3502.33 to 6422.82) | | 0.7820 | 0.000 | 0.3357809 | 0.4989451 |
| Mongolia | 1093.98 (852.41 to 1369.87) | 2037.92 (1602.84 to 2551.3) | | 1.9767 | 0.000 | 0.4665501 | 0.6176216 |
| Montenegro | 2627.6 (2026.98 to 3324.34) | 3914.59 (3026.97 to 4963.02) | | 1.4059 | 0.000 | 0.6742257 | 0.7958006 |
| Nepal | 3036.7 (2339.05 to 3908.05) | 4960.94 (3752.68 to 6469.32) | | 1.5607 | 0.000 | 0.1995607 | 0.4331746 |
| Nigeria | 4190.64 (3503.85 to 4954.87) | 4859.87 (3991.35 to 5883.76) | | 0.5069 | 0.000 | 0.3058680 | 0.5033908 |
| Poland | 2729.45 (2314.96 to 3246.49) | 3281.42 (2628.79 to 4064.32) | | 0.5117 | 0.000 | 0.6272279 | 0.8120428 |
| Republic of Moldova | 1552.95 (1254.85 to 1899.68) | 2461.8 (1916.13 to 3115.82) | | 1.6377 | 0.000 | 0.6042518 | 0.7322149 |
| Romania | 1613.81 (1300.56 to 1987) | 2028.98 (1563.29 to 2585.8) | | 0.7166 | 0.000 | 0.6192989 | 0.7684539 |
| Russian Federation | 1062.93 (867.74 to 1311.77) | 2878.08 (2479.86 to 3402.49) | | 2.9012 | 0.000 | 0.6716006 | 0.8085360 |
| Serbia | 3610.61 (2822.34 to 4518.41) | 4604.44 (3582.41 to 5834.36) | | 0.8392 | 0.000 | 0.6305110 | 0.7924163 |
| Sierra Leone | 3188.51 (2401.13 to 4126.93) | 4895.92 (3707.37 to 6433.98) | | 1.4199 | 0.000 | 0.2115693 | 0.3586659 |
| Slovakia | 2288.35 (1783.06 to 2852.68) | 2397.63 (1825.57 to 3085.22) | | 0.0763 | 0.587 | 0.6538535 | 0.8106105 |
| Sudan | 2225.33 (1718.7 to 2887.65) | 4059.04 (3095.79 to 5267.8) | | 1.9679 | 0.000 | 0.2921786 | 0.5419497 |
| Syrian Arab Republic | 3050.66 (2371.05 to 3855.49) | 4629.33 (3523.77 to 6072.68) | | 1.3153 | 0.000 | 0.4304926 | 0.6230041 |
| Tajikistan | 1849.47 (1505.58 to 2245.02) | 2829.74 (2232.63 to 3585.94) | | 1.3720 | 0.000 | 0.4661554 | 0.5415112 |
| Timor-Leste | 2651.13 (1967.47 to 3517.66) | 4646.54 (3489.33 to 6111.41) | | 1.9049 | 0.000 | 0.2624681 | 0.4446676 |
| Togo | 3031.37 (2254.38 to 4039.44) | 4690.24 (3455.43 to 6284.12) | | 1.4192 | 0.000 | 0.2696923 | 0.4085337 |
| Tunisia | 2190.5 (1695.46 to 2810.4) | 4728.59 (3521.34 to 6291.94) | | 2.4715 | 0.000 | 0.4711385 | 0.6824322 |
| Turkey | 4577.93 (3546.39 to 5786.63) | 4847.34 (3752.07 to 6138.94) | | 0.0568 | 0.641 | 0.4616070 | 0.7126927 |
| Turkmenistan | 1558.7 (1328.14 to 1805.82) | 3470.31 (2805.71 to 4347.55) | | 2.8094 | 0.000 | 0.5631269 | 0.6821608 |
| Ukraine | 967.52 (780.75 to 1198.59) | 1411.82 (1056.51 to 1847.8) | | 1.2487 | 0.000 | 0.6474615 | 0.7607739 |
| Uzbekistan | 1636.93 (1394.06 to 1903.92) | 4699.61 (3916.04 to 5650.83) | | 3.4367 | 0.000 | 0.5002417 | 0.6626217 |
| Yemen | 2088 (1558.21 to 2819.28) | 3330.8 (2487.48 to 4447.9) | | 1.4972 | 0.000 | 0.2156646 | 0.4503764 |

Table S8. In dual-burden regions, ASDR of type 2 diabetes mellitus among people aged 55 years and older in 1990 and 2021, and change from 1990 to 2021. Abbreviations: ASDR, age-standardized disability-adjusted life years rates; SDI, sociodemographic index; UI, uncertainty interval; AAPC, average annual percent change.

| Location | ASDR, per 100,000 (95% UI) | | | AAPC | P value | SDI 1990 | SDI 2021 |
| --- | --- | --- | --- | --- | --- | --- | --- |
|  | 1990 | | 2021 |  |  |  |  |
| Afghanistan | 4246.37 (3207.53 to 5689.89) | 8449.03 (6413.32 to 11101.02) | | 2.2746 | <0.001 | 0.1738322 | 0.3372000 |
| American Samoa | 12136.58 (9644.39 to 14833.48) | 16669.53 (13500.94 to 20339.93) | | 1.0803 | <0.001 | 0.6136339 | 0.7237275 |
| Angola | 6196.46 (4622.5 to 8232.43) | 6933.7 (5226.49 to 8893.7) | | 0.3320 | <0.001 | 0.2707369 | 0.4537219 |
| Bahrain | 13097.75 (10571 to 15882.49) | 17025.47 (13647.17 to 20673.09) | | 0.5141 | <0.001 | 0.5845789 | 0.7530432 |
| Bosnia and Herzegovina | 2826.04 (2234.62 to 3522.14) | 5798.45 (4550.11 to 7287.09) | | 2.3847 | <0.001 | 0.5411325 | 0.7230779 |
| Cabo Verde | 2019.01 (1573.23 to 2534.55) | 5761.77 (4473.13 to 7317.62) | | 3.6764 | <0.001 | 0.2767234 | 0.5335345 |
| Cambodia | 3714.82 (2822.33 to 4852.11) | 5296.87 (3979.24 to 6954.93) | | 1.1969 | <0.001 | 0.2890751 | 0.4736215 |
| Central African Republic | 7698.87 (5870.85 to 9905.23) | 8606.59 (6503.19 to 11253.7) | | 0.3696 | <0.001 | 0.2168252 | 0.3091677 |
| Congo | 7669.18 (5763.56 to 9932.42) | 8328.78 (6339.18 to 10809.16) | | 0.2883 | <0.001 | 0.4206547 | 0.5830752 |
| Coted'Ivoire | 4062.96 (3122.47 to 5274.43) | 5896.61 (4500.48 to 7682.51) | | 1.1694 | <0.001 | 0.2793204 | 0.4259419 |
| Democratic Republic of the Congo | 5999.71 (4445.74 to 7940.38) | 6641.67 (4897.61 to 8788.58) | | 0.3076 | <0.001 | 0.2898432 | 0.3831798 |
| Dominican Republic | 3647.38 (2941.84 to 4497.95) | 5951.63 (4543.88 to 7623.49) | | 1.6801 | <0.001 | 0.4426541 | 0.6193882 |
| Egypt | 3600.67 (2862.97 to 4446.98) | 8374.74 (6665.43 to 10465.72) | | 2.8606 | <0.001 | 0.4171827 | 0.6067871 |
| Equatorial Guinea | 6778.48 (4936.21 to 8961.84) | 8623.23 (6191.36 to 11654.26) | | 0.8554 | <0.001 | 0.2687836 | 0.6578575 |
| Eswatini | 9954.18 (7525.68 to 12889.61) | 15750.3 (11660.5 to 21026.25) | | 1.6151 | <0.001 | 0.3994210 | 0.5854597 |
| Fiji | 22544.01 (17973.9 to 27896.52) | 32197.24 (25247.59 to 40687.36) | | 1.1379 | <0.001 | 0.5346489 | 0.6750516 |
| Gabon | 7649 (5593.45 to 10355.13) | 9866 (7250.74 to 13132.11) | | 0.9047 | <0.001 | 0.4554212 | 0.6346914 |
| Gambia | 3482.6 (2606.8 to 4630.06) | 6032.11 (4487.82 to 8031.82) | | 1.8498 | <0.001 | 0.2387148 | 0.4097142 |
| Guinea | 3580.27 (2570.46 to 4855.26) | 5475.39 (4110.51 to 7231.35) | | 1.4121 | <0.001 | 0.1782954 | 0.3364013 |
| Guinea-Bissau | 5226.34 (3903.75 to 6824.33) | 7256.21 (5577.46 to 9350.97) | | 1.1118 | <0.001 | 0.2076148 | 0.3531096 |
| Guyana | 11227.99 (9655.23 to 13023.58) | 13872.75 (11219.04 to 17021.76) | | 0.8190 | <0.001 | 0.4604301 | 0.6508123 |
| Haiti | 11194.12 (8451.22 to 14353.8) | 11939.59 (8885.75 to 15777.09) | | 0.2017 | <0.001 | 0.3103346 | 0.4482783 |
| Honduras | 2952.95 (2323.16 to 3717.77) | 5899.85 (4498.77 to 7716.43) | | 2.3126 | <0.001 | 0.3320429 | 0.5130372 |
| Iraq | 6227.73 (4860.08 to 7936.92) | 9239.59 (7187.3 to 11802.35) | | 1.2785 | <0.001 | 0.4120442 | 0.6626262 |
| Kiribati | 16571.94 (12964.44 to 20792.92) | 22304.06 (17058.13 to 29177.46) | | 0.9453 | <0.001 | 0.4103898 | 0.5271866 |
| Kuwait | 4479 (3606.51 to 5533.3) | 6998.67 (5350.36 to 9120.85) | | 1.3922 | 0.011 | 0.6645179 | 0.8466511 |
| Lao People's Democratic Republic | 4811.44 (3558.35 to 6397.7) | 6116.17 (4648.79 to 7827.51) | | 0.7617 | <0.001 | 0.2642832 | 0.4891361 |
| Liberia | 3926.82 (2966.54 to 5111.53) | 6084.93 (4460.42 to 8126.29) | | 1.3086 | <0.001 | 0.2352968 | 0.3524425 |
| Libya | 2620.32 (2006.71 to 3320.01) | 5831.95 (4441.31 to 7651.87) | | 2.7286 | <0.001 | 0.5279982 | 0.7257714 |
| Marshall Islands | 13974.1 (10643.66 to 18189.69) | 22122.46 (16051.59 to 30293.57) | | 1.4972 | <0.001 | 0.4308393 | 0.5740911 |
| Micronesia (Federated States of) | 10712.76 (8067.35 to 13985.24) | 15694.31 (11998.71 to 20324.58) | | 1.2346 | <0.001 | 0.4625116 | 0.5875350 |
| Morocco | 2739.4 (2121.63 to 3543.89) | 6549.89 (4955.43 to 8560.06) | | 2.8943 | <0.001 | 0.3580729 | 0.5626983 |
| Myanmar | 7306.25 (5486.93 to 9431.11) | 8651.1 (6742.15 to 10939.34) | | 0.5123 | <0.001 | 0.3192197 | 0.5339008 |
| Nauru | 14404.17 (10747.26 to 19187.91) | 19303.2 (14693.41 to 24766.41) | | 0.9651 | <0.001 | 0.5391460 | 0.6251778 |
| Niue | 10744.97 (8330.77 to 13762.99) | 16864.1 (12845.88 to 21736.49) | | 1.4528 | <0.001 | 0.5875330 | 0.7262220 |
| North Macedonia | 3712.91 (2955.13 to 4569.95) | 5792.03 (4540.85 to 7264.8) | | 1.5156 | <0.001 | 0.6090261 | 0.7506297 |
| Northern Mariana Islands | 8195.68 (6339.48 to 10494.37) | 8904.96 (7087.58 to 10893.21) | | 0.0959 | 0.553 | 0.7085938 | 0.7715352 |
| Oman | 5663.19 (4189.7 to 7679.46) | 7422.96 (5757.38 to 9426.73) | | 0.7863 | <0.001 | 0.4292709 | 0.7733916 |
| Pakistan | 3674.21 (2920.87 to 4544.45) | 6641.8 (5337.77 to 8204.07) | | 1.9823 | <0.001 | 0.3104676 | 0.5040287 |
| Palau | 10921.5 (8363.46 to 13987.64) | 14725.53 (11400.42 to 18566.59) | | 0.9704 | <0.001 | 0.6629095 | 0.7540469 |
| Palestine | 6754.68 (5137.3 to 8821.07) | 8526.96 (6796.06 to 10519.59) | | 0.6511 | <0.001 | 0.4017922 | 0.6310117 |
| Papua New Guinea | 10716.12 (7661.79 to 14682.47) | 12244.01 (9327.74 to 15656.44) | | 0.4385 | <0.001 | 0.3106686 | 0.4177974 |
| Philippines | 3944.38 (3430.4 to 4520.31) | 5735.07 (4934.42 to 6727.63) | | 1.3521 | <0.001 | 0.5100118 | 0.6512193 |
| Qatar | 11042.71 (8415.94 to 14241.01) | 12492.62 (9619.7 to 16024.22) | | -0.1238 | 0.747 | 0.6512084 | 0.8468606 |
| Samoa | 9987.48 (7777.05 to 12700.43) | 13419.32 (10474.88 to 16917.93) | | 0.9905 | <0.001 | 0.4874914 | 0.5933928 |
| Saudi Arabia | 3832.31 (2887.11 to 4962.96) | 5855.89 (4561.66 to 7434) | | 1.3057 | <0.001 | 0.5389545 | 0.8151435 |
| Senegal | 4296.8 (3309.88 to 5439.12) | 7044.55 (5338.4 to 9110.85) | | 1.6550 | <0.001 | 0.2380476 | 0.4080542 |
| Solomon Islands | 9488.19 (6397.2 to 13211.37) | 13091.3 (9858.24 to 17012.81) | | 1.0426 | <0.001 | 0.3012172 | 0.4293603 |
| Tokelau | 10327.24 (7810.86 to 13479.34) | 13004.1 (10209.75 to 16439.16) | | 0.7363 | <0.001 | 0.5219424 | 0.6864256 |
| Tonga | 11498.16 (9118.33 to 14237.13) | 15115.31 (11822.3 to 19132.52) | | 0.8768 | <0.001 | 0.4918068 | 0.6263499 |
| Trinidad and Tobago | 16822.72 (15127.42 to 18623) | 14635.9 (11859.62 to 17993.08) | | -0.4824 | 0.001 | 0.6239702 | 0.7687633 |
| Tuvalu | 10552.84 (7942.95 to 14047.15) | 13271.61 (10199.24 to 16959.69) | | 0.7823 | <0.001 | 0.4062476 | 0.5766205 |
| United Arab Emirates | 6442.74 (4861.29 to 8517.25) | 7208.98 (5480.23 to 9218.35) | | 0.6931 | 0.167 | 0.6444123 | 0.8493177 |
| Vanuatu | 8753.39 (6310.26 to 12620.9) | 12022.03 (9306.95 to 15303.7) | | 1.0136 | <0.001 | 0.3531003 | 0.4731007 |
| Venezuela (Bolivarian Republic of) | 4937.18 (4319.7 to 5632.17) | 6712.28 (5387.59 to 8271.95) | | 0.9298 | <0.001 | 0.5168912 | 0.5965131 |
| Zimbabwe | 4766.59 (3664.21 to 6111.4) | 8958.91 (6747.43 to 11717.76) | | 2.1637 | <0.001 | 0.3985593 | 0.4738195 |
